# Towards a Point-of-Care (POC) Diagnostic Platform for the Multiplex Electrochemiluminescent (ECL) Sensing of Mild Traumatic Brain Injury (mTBI) Biomarkers

**DOI:** 10.3390/bios12030172

**Published:** 2022-03-11

**Authors:** Milica Jović, Denis Prim, Edis Saini, Marc Emil Pfeifer

**Affiliations:** Diagnostic Systems Research Group, Institute of Life Technologies, School of Engineering, University of Applied Sciences and Arts Western Switzerland (HES-SO Valais-Wallis), 1950 Sion, Switzerland; milica.jovic@hevs.ch (M.J.); denis.prim@hevs.ch (D.P.); edis.saini@lonza.com (E.S.)

**Keywords:** biomarker panel, biosensor, electrochemiluminescence (ECL), electrochemiluminescence immunoassay (ECLIA), mild traumatic brain injury (mTBI), multiplex assay, point-of-care (POC) diagnostics, sandwich immunoassay, screen-printed electrode (SPE)

## Abstract

Globally, 70 million people are annually affected by TBI. A significant proportion of all TBI cases are actually mild TBI (concussion, 70–85%), which is considerably more difficult to diagnose due to the absence of apparent symptoms. Current clinical practice of diagnosing mTBI largely resides on the patients’ history, clinical aspects, and CT and MRI neuroimaging observations. The latter methods are costly, time-consuming, and not amenable for decentralized or accident site measurements. As an alternative (and/or complementary), mTBI diagnostics can be performed by detection of mTBI biomarkers from patients’ blood. Herein, we proposed two strategies for the detection of three mTBI-relevant biomarkers (GFAP, h-FABP, and S100β), in standard solutions and in human serum samples by using an electrochemiluminescence (ECL) immunoassay on (i) a commercial ECL platform in 96-well plate format, and (ii) a “POC-friendly” platform with disposable screen-printed carbon electrodes (SPCE) and a portable ECL reader. We further demonstrated a proof-of-concept for integrating three individually developed mTBI assays (“*singleplex*”) into a three-plex (“multiplex”) assay on a single SPCE using a spatially resolved ECL approach. The presented methodology demonstrates feasibility and a first step towards the development of a rapid POC multiplex diagnostic system for the detection of a mTBI biomarker panel on a single SPCE.

## 1. Introduction

Traumatic brain injuries (TBI) are physical injuries that can lead to brain function alterations of temporary or permanent nature [1]. In the absence of routine diagnostic screening tests for TBI, the number of truly affected patients is difficult to assess and is probably significantly under-reported [2]. According to the CDC statistics, 1.5 million people suffer from TBI each year, while the most frequent causes of TBI are motor vehicle crashes, falls, and violence [3]. The Glasgow Coma Scale (GCS) developed in the seventies of the 20th century, is an assessment tool used to determine the consciousness level of the patient after a TBI, based on the ability to open eyes, be oriented, respond to questions, and obey motor commands [4]. Based on the extent of injury, TBIs can be classified as mild (GCS 13–15), moderate (GCS 9–12), and severe (GCS 3–8). A vast majority of TBI cases can be attributed to concussion, i.e., mild TBI [5]. Post-injury, there is often an absence of symptoms or primarily the presence of non-specific symptoms (e.g., headaches, fatigue, depression, visual and/or sleep disturbances, seizures, etc.) [6].

Diagnosis of mTBI can be rather challenging. The GCS scale is dependent on the assessment skills of the observer, and it can be inaccurate in distinguishing between mild and moderate TBIs [7]. Alternatives are neuroimaging tools, such as MRI and CT scans, which can be quite costly, expose patients to harmful radiation, and are not available on-site (accident sites, sports fields, remote and underdeveloped areas, etc.). Also, approximately 90% of all mTBI cases will not have any evidence of structural abnormalities visible on a CT scan [8]. Therefore, there is a growing need to develop additional diagnostic screening tools for aiding diagnosis and enabling an accurate, inexpensive and fast triage of patients with mTBI [8,9,10,11,12].

Biomarkers may have different intended uses, such as diagnostic, prognostic, predictive, pharmacodynamic, or efficacy response relevance. As for mTBI, the preferred ones are brain protein biomarkers that can pass the blood—brain barrier (BBB) into circulation. They can be found in human serum and/or plasma, and the analytical tools should be able to accurately detect them in the lower picogram range (pg mL^−1^) [13]. The Scandinavian guideline proposes the use of the biomarker S100 calcium-binding protein β (S100β) for mTBI patients who are admitted to the hospital within 6 h after the injury [14]. However, S100β can be increased in multiple extra-cerebral tissues, for example, after extracranial injuries [15], peripheral lesions [16], and physical exercise [17].

Recently, several publications indicated other mTBI biomarkers that could be more suitable. For example, glial fibrillary acidic protein (GFAP) is a protein found in the cytoskeleton of glial cells [18] and has been used for the detection of acute intracranial injuries following a TBI [19]. The sensitivity of GFAP for the detection of intracranial lesions on CT scans has been reported to be 67% to 100%, with specificity between 0% and 100% [20]. GFAP could be useful for differentiating focal and diffuse brain injury; however, its ability to enter into the bloodstream depends on the BBB damage [21]. Furthermore, heart fatty-acid binding protein (h-FABP) is a cytosolic trafficking protein that can predict intracranial pathologies linked with brain injuries [22,23].

TBI biomarkers have the potential to significantly advance the way TBI is assessed and treated today, beyond the evaluation of the need for a head CT scan [24]. However, given the complexity of the TBI, it is unlikely that any single biomarker would be sufficiently sensitive and specific for use as a clinical diagnostic test. Posti et al. and Lagerstedt et al. reported on panels of protein biomarkers that perform better in discriminating CT positive from CT negative patients with mTBI than individual biomarkers [25,26]. As early as the beginning of 2018, a central laboratory test developed by Banyan Biomarkers was approved by the FDA [27]. The test is based on a chemiluminescent ELISA format for determining two TBI biomarkers, GFAP and ubiquitin carboxyl-terminal hydrolase isozyme L1 (UCH-L1), with a sensitivity of 97.5% for CT positive cases and a negative predictive value of 99.6% [27]. Practically the latter means that in more than 33% of the cases, the patients being suspected of brain injury can be ruled out prior to a CT scan [27]. Just recently, in January 2021, Abbott launched the POC diagnostic device i-STAT^TM^ Alinity^TM^ that measures amperometrically UCH-L1 and GFAP [28], while the company NanoDx™ is in the progress of developing an ultrasensitive nanowire technology to resistively measure the S100β and GFAP biomarkers [29].

**Scheme 1:**
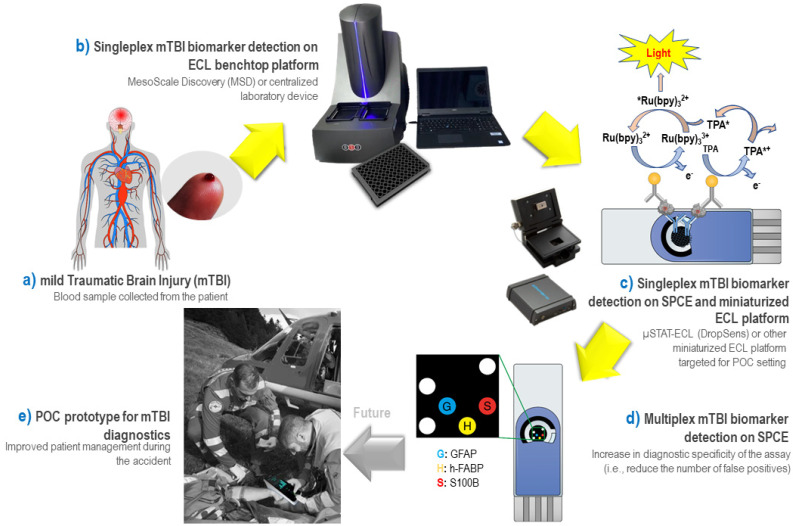
Schematic representation of a strategy proposed for the detection of blood proteins for mild traumatic brain injury (mTBI) diagnostics: (**a**) Illustration of the human body suffering from an mTBI injury and collection of blood sample potentially containing mTBI-relevant protein biomarkers. (**b**) Development of ECL-based sandwich immunoassay for each individual mTBI-relevant biomarker (“*singleplex*”) on a benchtop MesoScale Discovery (MSD) platform in 96-well plate format: (**c**) Translation of developed ECL detection strategy for each individual mTBI-relevant biomarker (“*singleplex*”) from benchtop MSD platform into a miniaturized “apropos Point-Of-Care (POC)” ECL platform based on screen-printed carbon electrodes (SPCE) and µSTAT-ECL reader from DropSens Metrohm. (**d**) Proof-of-concept for a multiplex ECL detection of three different mTBI-relevant biomarkers (i.e., GFAP, h-FABP and S100β) on a single SPCE using a spatially resolved approach and a charge-couple device (CCD) camera as ECL detector. (**e**) Illustration of the envisioned POC diagnostic instrument in the context of medical emergencies allowing rapid treatment of patients following the accident. Steps (**b**–**d**) are achieved in the context of the present publication, while the step (**e**) represents the “ultimate” goal of the proposed methodology being the integration of the developed assays into the POC prototype for decentralized mTBI diagnostics (undeveloped areas, emergency rooms, battlefield, sports facilities, car accident sites, etc.).

Despite the recent developments in the mTBI diagnostic field, there is still a growing need to expand the biomarker panel with inflammatory and brain damage biomarkers to guarantee adequate diagnostic specificity and sensitivity and to allow decentralized mTBI diagnostics. To address these limitations, the conception of a minimally invasive multiplexed detection device would allow rapid and reliable detection of mTBI biomarkers at the POC level, which could revolutionize mTBI-patients management compared to conventional methods used. Many different electrochemical (EC) (bio)sensors approaches [30] and few ECL biosensor approaches have been proposed for detection of single, individual mTBI biomarkers (h-FABP [31,32]), but to the best of our knowledge, none using a mTBI biomarker panel. Electrochemiluminescence immunoassays (ECLIA) show great promise for the detection of low-level concentration compounds, with a wide linear range, high sensitivity, and low background noise [33]. In this work, we have developed a sandwich-type ECL assay for detecting and quantifying three mTBI-relevant biomarkers (GFAP, h-FABP and S100β, Table 1), in standard solutions and in human serum samples (HS), using a 96-well plate commercial “benchtop” platform from MesoScale Discovery (MSD) (Scheme 1b). These three individual “*singleplex*” assays were then translated into a miniaturized platform based on screen-printed carbon electrodes (SPCE) coupled with a portable ECL reader (µSTAT-ECL DropSens Metrohm, Scheme 1c). Furthermore, we showed a proof-of-concept for integration of three individually developed mTBI biomarker assays (“*singleplex*”) into a three-plex “multiplex” assay on a single SPCE using a spatially resolved ECL approach (Scheme 1d), demonstrating a first step towards the development of a POC diagnostic prototype instrument based on ECL detection of a mTBI biomarker panel (Scheme 1e).

## 2. Materials and Methods

### 2.1. Materials

GFAP assay: Antigen GFAP human recombinant (ref. 8G45, HyTest Ltd., Turku, Finland); monoclonal mouse anti-human glial fibrillary acidic protein (ref. 4G25, HyTest Ltd., Turku, Finland) clone 83cc and clone 81cc were employed as capture and detection antibody, respectively.

h-FABP assay: Antigen FABP human (ref. 8F65, HyTest Ltd., Turku, Finland); monoclonal mouse anti-human fatty acid-binding protein (ref. 4F29, HyTest Ltd., Turku, Finland) clone 22 and clone 28cc were employed as capture and detection antibody, respectively.

S100β assay: Antigen S100BB homodimer and S100A1B heterodimer human (ref. 8S9h, HyTest Ltd., Turku, Finland); monoclonal mouse anti-human S100 proteins (ref. 4S37, HyTest Ltd., Turku, Finland) clone 8B10cc and clone 6G1cc were employed as capture and detection antibody, respectively.

Serum samples: Human serum from human male AB plasma, US origin, sterile filtered (ref. H4522, Sigma Aldrich, St. Louis, MO, USA) was diluted 2× with appropriate assay diluents and used for the recovery studies.

Other reagents: All chemicals were used as received without further purification. All aqueous solutions were prepared with MQ water. Gold SULFO-Tag NHS-Ester lyophilized (ref. RA19AO), read buffer T 4X (ref. R92TC), conjugation buffer (ref. R60AJ), conjugation storage buffer (ref. R60AC) and QuickPlex 96-well plate (ref. L55XA) were all purchased from Meso Scale Discovery (MSD). Other materials included: Zeba Spin desalting columns 40K MWCO 0.5 mL (87766, ThermoFisher, Waltham, MA, USA), Millex-GV Filter 0.22 µm (SLGV004SL, Sigma-Aldrich, MO, USA), syringe 1 mL BD Luer-Lok tip (309628, BD, New York, NJ, USA), casein sodium salt from bovine milk (C8654-500G, Sigma-Aldrich, MO, USA), bovine serum albumin fraction V (ref. 10735078001, Roche Diagnostics, Rotkreuz, Switzerland), PBS 10× pH 7.4 phosphate saline buffer (ref. 7011-044, Gibco, Billings, MT, USA), Tween-20 (ref. P1379-100 mL, Sigma-Aldrich, MO, USA), CaCl_2_·2H_2_O (ref. 223506, Fluka, Buchs, Switzerland). Technical grade ethanol was used for cleaning SPCE electrodes.

### 2.2. Apparatus

QuickPlex SQ120 (Meso Scale Discovery MSD): The MSD platform is based on ECL detection of a SULFO-Tag labelled detection antibody that emits light upon electrochemical stimulation. QuickPlex SQ120 was employed as an ECL plate reader for QuickPlex 96-well plates. The detection process is initiated on carbon electrodes located at the bottom of the wells, and only labels in the electrode proximity can be detected. The ECL mechanism of the co-reactant system ruthenium tris(bipyridine)-tripropylamine (read buffer) has been previously described [40].

Disposable screen-printed electrodes (SPEs, Metrohm DropSens): The SPEs incorporate a three-electrode setup, printed on ceramic substrates (size 33.0 mm × 10.0 mm). Both working (WE; disk-shaped 4-mm diameter) and counter-electrodes are fabricated from carbon or carbon-based inks (for electrodes with modified WE), while pseudo-reference electrodes and electrical contact pads are fabricated from silver ink. An insulating layer is printed over the three-electrode system, leaving the electric contacts and a working area with an actual volume of 50 μL. SPEs with different types of working electrodes were tested as the solid-state support throughout this work: screen-printed carbon electrodes (SPCE, ref. DRP-110), screen-printed carbon electrodes modified with gold nanoparticles (SPCE-GNP, ref. DRP-110-GNP), screen-printed carbon electrodes modified with carbon nanotubes and gold nanoparticles (SPCE-CNT-GNP, ref. DRP-110CNT-GNP) and screen-printed carbon electrodes modified with quantum dots (SPCE-QD, ref. DRP-110QD).

Homemade incubation cell for SPEs: Customized incubation cell for SPEs was fabricated from Teflon at the HES-SO Valais-Wallis mechanical workshop (Supplementary Figure S6). The cell was designed using the SolidWorks CAD package and was intended to fit 12 SPEs, leaving only the WE area to be exposed to the reagents during various incubation/mixing/washing steps of the immunoassay protocol. When the immunoassay protocol was finished, the SPEs were taken out of the incubation cell, washed with wash buffer solution, and transferred into µSTAT-ECL cell for the read-out.

µSTAT Bipotentiostat with ECL Cell (Metrohm DropSens): The device is composed of a potentiostat/galvanostat (±4 V DC potential range, ±40 mA maximum measurable current) (size 127.8 mm × 124.1 mm × 34.1 mm) combined with a detector that is integrated in the ECL cell (size 75.0 mm × 88.4 mm × 40.0 mm). The detector is a low-noise photosensor composed of a silicon photodiode with a spectral response range of 340–1100 nm, and a maximum sensitivity of 0.62 V/nW at 960 nm [41]. The device uses the DropView 8400 software for signal acquisition and can be employed as a portable, battery-operated ECL reader.

Multiplex electrode spotting with nano-spotter device: A S3 contactless nano-spotting device from Scienion AG (Berlin, Germany) equipped with a Piezo Dispense Capillary (PDC-70, coating type 3, p/n: P-2030 S-6051) was used to dispense drops of 300 pL on pre-defined positions of Metrohm DropSens electrodes.

Multiplex assay measurement imager: For ECL read-out from multiplex assay developed on SPE electrodes, a Vilber Fusion FX6 EDGE imager (Vilber Smart Imaging) was employed in combination with a µSTAT Bipotentiostat. The imager is equipped with an eVo6 scientific grade CCD camera (6.3 MPx, −30 °C cooling, f/0.7) to acquire signals generated from SPE. ImageJ software (v 1.53) was used for image processing.

### 2.3. Methods

Detection antibody conjugation with Ru(bpy)_3_^2+^-NHS ester: Detection antibody labeling with Gold SULFO-Tag NHS-Ester was performed following the protocol provided by MesoScale Discovery (MSD GOLD SULFO-TAG Conjugation Quick Guide). Challenge ratio of 50:1 was used for all detection antibodies, while the labeling incorporation ratio was calculated based on the OD_455_ measured for each labelled antibody using the NanoDrop OnceC Microvolume UV-VIS Spectrophotometer (Thermo-Fisher Scientific, Waltham, MA, USA). The calculated label ratio was 19:1, 14:1 and 21:1 for GFAP, h-FABP and S100β detection antibodies, respectively.

Singleplex assay on MSD platform: 30 µL of capture antibody (at desired concentration, diluted in the appropriate coating diluent) was added in the well of QuickPlex 96-well plate. After the incubation step (1.5 h, room temperature RT, 700 rpm) the plate was washed using a dedicated wash buffer (3 × 300 µL), and 100 µL of blocking agent was added (45 min, RT, 700 rpm). After the second washing step, 30 µL of antigen or blank was added in the well and incubated (1 h, RT, 700 rpm). After the third washing step, 30 µL of detection antibody was added at desired concentration (diluted in corresponding diluent) and incubated (1 h, RT, 700 rpm). After the final washing step, 150 µL of MSD read buffer 2× was added in each well and ECL signal was recorded using MSD QuickPlex SQ120 plate reader.

Singleplex assay on SPCEs: SPCEs were firstly washed using the mixture of ethanol/water (2:1) for 20 min, then rinsed with DI water, dried with nitrogen, and placed inside the “homemade” incubation cell. The immunoassay protocol was identical as described in the paragraph above for MSD platform, with the difference that all incubation steps were performed at 500 rpm. After the last incubation/washing step, the SPCEs were taken out from the incubation cell and placed inside the ECL cell of µSTAT-ECL instrument with 50 µL of MSD read buffer 2× to perform the read-out.

Multiplex assay on SPCEs: Electrodes were cleaned using the same protocol as described for the *singleplex* assay. The multiplex assay was performed using the assay conditions established for S100β biomarker (Table 2). The capture antibodies (50 µg mL^−1^) were deposited on the working electrode using the automated nano-spotter device (S3, Scienion, Berlin, Germany) by collocated spotting of 30 drops of 300 pL (±10 pL), to form spots of 250 µm (±50 µm) diameter. The source plate temperature was set at RT and the relative humidity in the spotting area at 60%. After deposition, SPCEs were let in the spotting area during 30 min before blocking with 2% BSA for 1 h at RT and washing with wash buffer. Incubation with antigen and detection antibodies was carried out in homemade incubation cells for SPEs. The read-out was performed with 150 µL of MSD read buffer 2× using Vilber Fusion FX6 EDGE imager (Vilber Smart Imaging) combined with µSTAT Bipotentiostat.

## 3. Results

### 3.1. Development of ECL “Singleplex” Assays for mTBI Biomarkers on MSD Platform

MSD QuickPlex SQ120 is a versatile and robust platform that can be very useful tool for the detection of different types of analytes in 96-well plate format, and it was employed for development of ECL sandwich immunoassays for each of the individual mTBI biomarkers (GFAP, h-FABP and S100β) (Scheme 1b).

A design of experiment (DoE) approach was used to determine the optimal settings and conditions for the major controllable factors in the assay. The following conditions were jointly assessed for development/optimization of each individual mTBI biomarker assay:

- Coating diluent (PBS 1× pH 7.4 or TRIS 50 mM pH 8.6, with addition of 0.1–5 mM CaCl_2_ for S100β assay)
- Blocking agent (0.1–2% of BSA or 0.1–2% casein in PBS 1× pH 7.4 or TRIS 50 mM pH 8.6, with/without addition of 0.1% Tween-20);
- Wash buffer (PBS 1× pH 7.4 or TRIS 50 mM pH 8.6, with 0.05–0.4% Tween-20);
- Detection antibody diluent (PBS 1× pH 7.4 or TRIS 50 mM pH 8.6, with/without addition of 0.1–1% of blocking agent and/or 0.06% Tween-20);
- Capture antibody (cAb) and detection antibody concentration (dAb) (cAb concentration range: 1–25 μg mL^−1^; dAb concentration range: 0.5–10 μg mL^−1^).

Table 2 summarizes the pre-optimized assay conditions obtained for each individual mTBI biomarker. In overall, the optimized assay conditions for GFAP and h-FABP assay were very similar. The optimal blocking agent was 1% BSA and all diluents were based on PBS 1×. In the case of the S100β assay, all diluents were based on 50 mM TRIS buffer (pH 8.6) with the addition of CaCl_2_, due to the fact that the S100 protein is a dimeric member of the EF-hand calcium-binding protein superfamily, and its calcium-binding properties influencing the antibody recognition [42,43]. Several studies indicated that such interaction happens through a calcium-induced conformational change, which leads to the exposure of a hydrophobic protein region [42].

**Figure 1:**
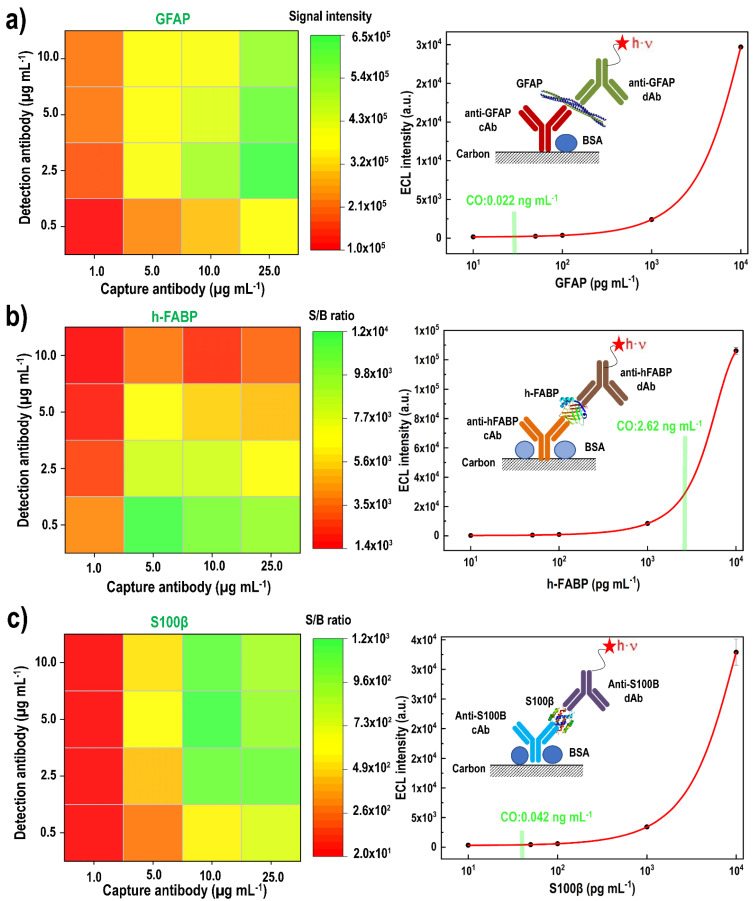
ECL sandwich immunoassays for mTBI-relevant biomarkers on the MSD platform in a 96-well plate format: (**a**) GFAP, (**b**) h-FABP, and (**c**) S100β. Left figures—Optimization of antibody concentrations in the sandwich assay: checkerboard optimization assay for the antibody pairs with the *x*-axis representing the capture antibody concentrations and the *y*-axis the detection antibody concentrations. The results are visualized as a heat map plot with reported signal intensities or S/B ratios (100 ng mL^−1^/blank). Right figures—ECL calibration curves established using the conditions listed in Table 2. The error bars represent the standard deviation from three replicates; bars are smaller than the data symbol employed. CO—cutoff value for the biomarker.

In some publications, the authors reported that the addition of Ca^2+^ in the antibody diluents had as a consequence, an improvement of the recognition activity [44,45], while others reported that calcium-chelators improved the antigen recognition (immunoassays with Sangtec antibodies) [46]. In the present study, we noticed that the addition of Ca^2+^ in the coating, assay and detection antibody diluent had a positive impact on the assay performance. This is also supported by the fact that the antibody provider recommends add it the ion of Ca^2+^ in the antibody diluents. Concentrations of capture and detection antibody were optimized for each biomarker using the mean matrix heatmap format. The results are presented in Figure 1 (left figures) as a heat map showing the signal intensities (GFAP assay) and S/B ratios (h-FABP and S100β assay) from low (red) to high (green). The capture and detection antibody concentrations were determined based on the maximum S/B results, apart from GFAP assay, where the antibody concentrations were chosen based on the highest signal intensities.

To evaluate the analytical performance of each *singleplex* biomarker assay, buffer solutions containing each individual biomarker concentration ranging from 10 pg mL^−1^ to 10 ng mL^−1^ were analyzed. Based on the obtained results, a calibration curve (Figure 1, right figures) was established for each biomarker using the pre-optimized conditions from Table 2. Data were analyzed by assuming that the ECL intensity was proportional to the biomarker concentration through a four-parameter dose-response regression function (4PL) model with 1/Y^2^ weighting (OriginPro software). To fit the data, the following equation was used (Equation (1)):

(1) $$y=\frac{A_{1}-A_{2}}{1+{\left(\frac{x}{x_{0}}\right)}^{p}}+A_{2}$$

where *x* denotes the concentration of the biomarker, and *A*_1_, *A*_2_, *x*_0_, and *p* are the four parameters. The *A*_1_ and *A*_2_ parameters correspond to the upper and lower asymptotes for the function, respectively, while the *p* (Hill slope) and *x*_0_ parameters correspond to the slope.

As Figure 1 indicates, the developed *singleplex* assays had good dynamic ranges, background levels, and sensitivities. The LOD values were calculated by interpolating the curve using the average value of the blank plus three times the standard deviation of the blank. Obtained LOD values of 6.94 pg mL^−1^ (R^2^ = 0.9999), 1.35 pg mL^−1^ (R^2^ = 0.9999) and 15.73 pg mL^−1^ (R^2^ = 0.9999) were achieved for GFAP, h-FABP and S1ooβ biomarker, respectively.

Once the suitability for mTBI biomarker panel detection has been established on the MSD platform, the assays were translated to SPE-based ECL test set-up platform.

### 3.2. Development of ECL “Singleplex” Assays for mTBI Biomarkers on SPE

#### 3.2.1. Choice of Electrode Material

The use of SPE in the context of highly sensitive ECL analytical and electrochemical diagnostic applications requires several different electrode characteristics such as: (a) fast electron transfer; (b) reproducible electrode surfaces that can improve the assay accuracy and precision; (c) large electroactive electrode areas to boost signal intensities; (d) broad potential window; and (e) a hydrophobic electrode surface to facilitate TPA oxidation on the electrode surface [47,48,49]. Hence, four commercially available SPEs were investigated for an application in ECL detection of mTBI biomarkers: SPCEs (DRP-110), SPCEs modified with gold nanoparticles SPCE-GNP (DRP-110-GNP), SPCEs modified with carbon nanotubes and gold nanoparticles SPCE-CNT-GNP (DRP-110CNT-GNP), and SPCEs modified with quantum dots SPCE-QD (DRP-110QD).

Firstly, the electrochemical properties of each electrode were tested using a standard redox couple (ferri/ferrocyanide) (Table 3). All electrodes showed quasi-reversible electrochemical behavior with the peak-to-peak separation (ΔE) higher than anticipated for the one-electron transfer process (>59 mV). The values were consistent with the ones reported by Banks et al. [50] and Fanjul-Bolado et al. [51] stating that it arises from a combination of the electrode properties (electrode material, composition of the paste used for the fabrication, the curing temperature, the hydrophilic characteristics of the electrode surface) [49]. The electrode electro-active area (A) was calculated using the Randles–Sevcik equation (Equation (2)) by studying the scan rates of 5 mM K_4_[Fe(CN)_6_]/K_3_[Fe(CN)_6_] in 0.1 M PBS) [52]:

i_p_ = (2.69 × 10^5^) · n^3/2^ · A · C · D^1/2^ · ν^1/2^ (2)

i_p_ is the peak current (A), n is the number of electrons involved in the redox reaction, A is the active electrode area (cm^2^), C is the concentration of redox molecule (mol/cm^3^), D is the diffusion coefficient (cm^2^/s), and ν is the scan rate (V/s).

The representative cyclic voltammograms showing the scan rate studies with ferri/ferrocyanide couple on different SPEs are shown in Figure S2a. SPCE and SPCE-GNP electrodes showed the highest active electrode area (A) and the highest conductivity (the lowest peak-to-peak separation, ΔE). Electrochemical properties of each electrode material were also tested with [Ru(bpy)_3_]^2+^ in PBS 1×. All CVs showed a reversible oxidation peak at ~0.5 V vs. Ag and a corresponding reduction peak at ~0.3 V vs. Ag pseudo-reference electrode (illustrated in Figure S2b).

**Figure 2:**
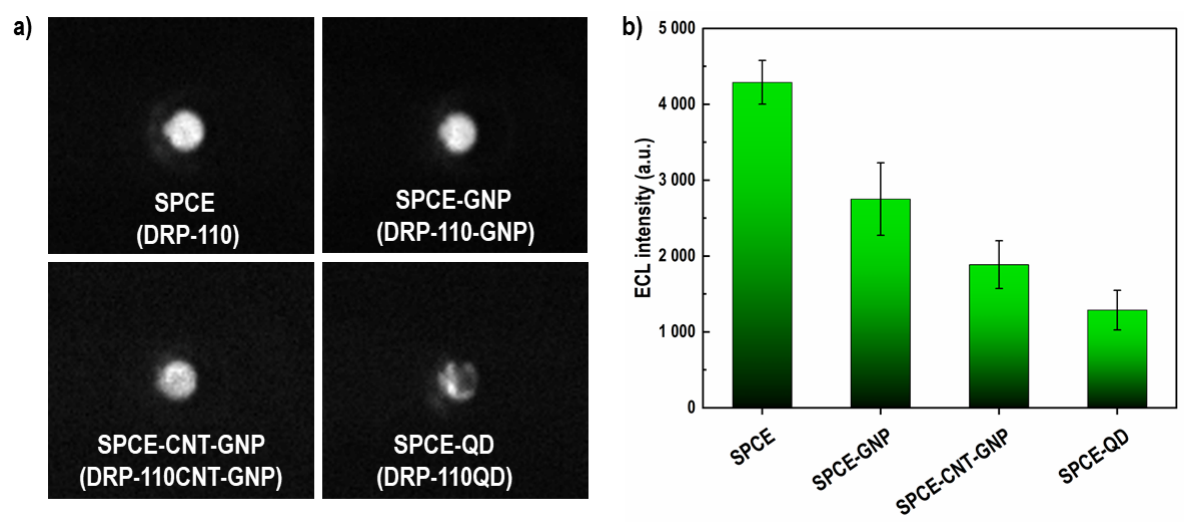
(**a**) Representation of ECL images obtained from four different commercial screen-printed electrodes (SPEs) using Vilber Fusion FX6 EDGE (Vilber Smart Imaging) (MSD Free Tag solution 15,000 a.u., 100 µL, LSV 200 mV/s, 0.1–1.8 V); (**b**) ECL intensity (n = 3) calculated by integration of light spot using ImageJ software, errors bars correspond to STDEV. Electrodes tested: SPCE (DRP-110), SPCE-GNP (DRP-110-GNP), SPCE-CNT-GNP (DRP-110CNT-GNP), and SPCE-QD (DRP-110QD).

Furthermore, to be able to compare the SPEs in terms of relative ECL intensities, a commercial solution containing [Ru(bpy)_3_]^2+^ and TPA (MSD Free Tag 15,000) was used to generate ECL signal upon applying the potential on SPEs (Figure 2). In that context, SPCE electrodes showed the highest ECL intensities, followed in decreasing order by SPCE-GNP > SPCE-CNT-GNP > SPCE-QD.

Since SPCEs showed the best electrochemical performance and exhibited the highest ECL intensity (Figure 2), they were selected as solid-state support for development of mTBI assays. SEM images of SPCE electrodes are shown in Figure S3.

#### 3.2.2. Optimization of SPCE-Based ECL Sensor

**Figure 3:**
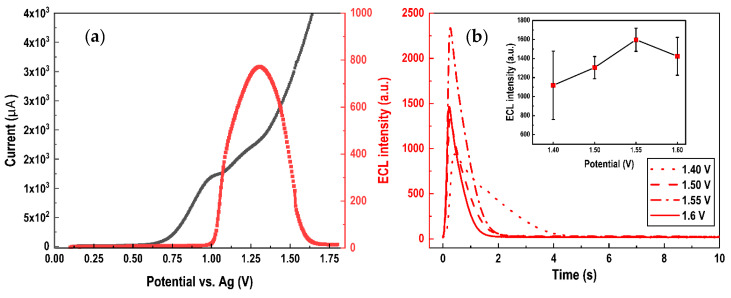
(**a**) Linear sweep voltammogram of SPCE for h-FABP biomarker assay (scan rate 200 mV/s) (black curve) and generated ECL signal (red curve). (**b**) ECL intensity curves from h-FABP biomarker assay on SPCE generated by chronoamperometry at different potentials: 1.4; 1.5; 1.55 and 1.6 V. Acquisition time 10 s. h-FABP assay conditions in Table 4 below (antigen concentration 100 ng mL^−1^).

The ECL signal of the co-reactant couple Ru(bpy)_3_^2+^–TPA is produced via the reaction between the deprotonated TPA radical (TPA^•^) and electrogenerated Ru(bpy)_3_^3+^, forming a [Ru(bpy)_3_^2+^] * radical that generates light emission when returning to the ground state (Equations (3)–(6)). TPA can be created via catalytic oxidation by electrogenerated Ru(bpy)_3_^3+^ (Equation (4a)) and direct electrode oxidation (Equation (4b)) [53]. Figure 3a shows an exemplary linear sweep voltammetric curve of SPCE (black line) and generated ECL curve (red line) obtained for h-FABP assay indicating a strong ECL peak at ~1.3 V vs. Ag electrode.

Ru(bpy)_3_^2+^ -> Ru(bpy)_3_^3+^ + e^−^ (3)

Ru(bpy)_3_^3+^ + TPA -> Ru(bpy)_3_^2+^ + TPA (4a)

TPA -> TPA^•^ + e^−^ (4b)

Ru(bpy)_3_^3+^ + TPA^•^ -> Ru(bpy)_3_^2+^ * (5)

Ru(bpy)_3_^2+^ * -> Ru(bpy)_3_^2+^ + hν (610 nm) (6)

Furthermore, two options were evaluated for the ECL signal trigger from SPCE: for (i) linear sweep voltammetry (LSV) (Figure 3a), versus (ii) constant potential chronoamperometry (CPA) (Figure 3b). For LSV, the scan rate of 200 mV/s for LSV has been selected considering that higher scan rates led to broadening and shift of ECL peak towards positive potentials, which consequently led to water oxidation, causing the formation of bubbles on the electrode surface that had a negative impact on detected ECL signals. For CPA three different potentials were tested: 1.4, 1.5, 1.55, and 1.6 V.

ECL signals generated by LSV were ~800 a.u. (Figure 3a), while for CPA it was found that the ECL signal intensity increased as the applied potentials became more positive, showing the maximum value of ~2250 a.u. at 1.55 V vs. Ag electrode (Figure 3b—inset plot). Based on these results it was decided to use CPA for the further experiments and development of mTBI biomarker assay on SPCE electrodes.

#### 3.2.3. Optimization of the Assay Conditions

**Figure 4:**
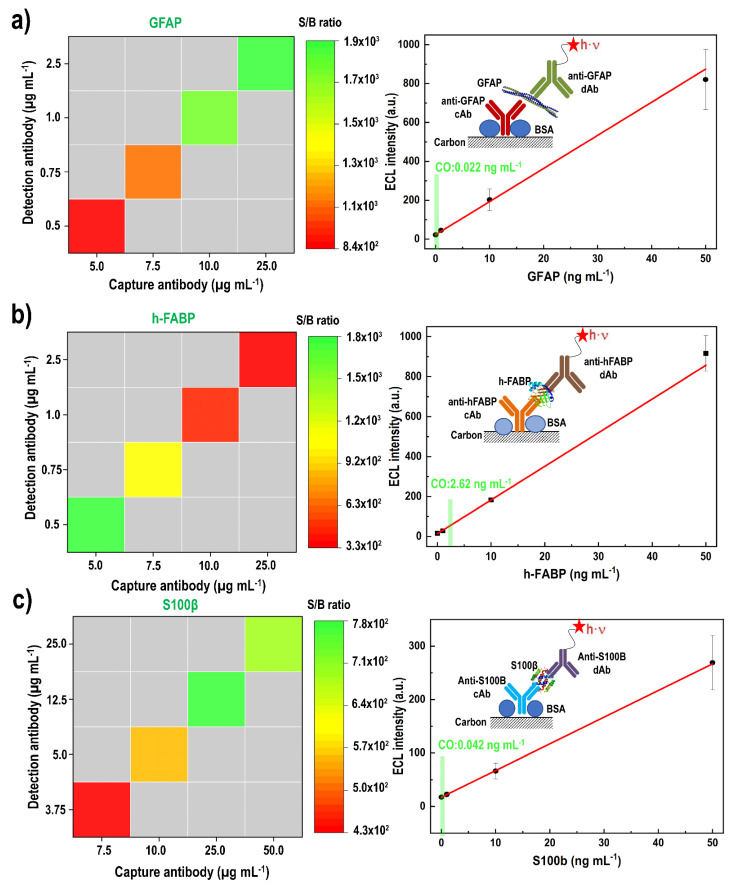
ECL immunoassays for mTBI-relevant biomarkers on SPCE µSTAT-ECL platform: (**a**) GFAP, (**b**) h-FABP, and, (**c**) S100β. Left figures–Optimization of antibody concentrations in the assay: checkerboard optimization assay for the antibody pairs with the *x*-axis containing the capture antibody concentrations and the *y*-axis the detection antibody concentrations (capture/detection antibody ratio 10/1). The results are presented as a heat map plot with reported S/B ratios (100 ng mL^−1^/blank). Right figures—ECL calibration curve established using the conditions listed in Table 4. The error bars represent the standard deviation from three replicates (n = 3); bars are smaller than the data symbol employed.

The pre-optimized assay conditions obtained for each mTBI biomarker on the MSD platform (Table 2) were translated to the SPCE and µSTAT-ECL platform, with the only difference that the concentrations of capture and detection antibodies were re-optimized using the checkerboard assay due to the differences in the WE area and morphology between MSD and SPCE electrodes (cAb-dAb ratio was kept constant, Table 4). The results are presented in Figure 4 (left figures) as a heat map showing the S/B ratios from low (red) to high (green). The optimal capture and detection antibody concentrations were determined based on the maximum S/B results and summarized in Table 4.

Analytical performance of the developed *singleplex* mTBI assays on the SPCEs was evaluated using the buffer samples containing each individual biomarker concentration ranging from 1 ng mL^−1^ to 50 ng mL^−1^ using pre-optimized conditions from Table 4. Based on the measured values, a calibration curve (Figure 4) was modeled by the linear regression equations for each mTBI biomarker (n = 3) (Figure S4). The LOD values were calculated using the average value of the blank and adding to it three times the standard deviation of the blank. LOD values of 0.59 ng mL^−1^ (R^2^ = 0.9777), 0.44 ng mL^−1^ (R^2^ = 0.9931) and 1.34 ng mL^−1^ (R^2^ = 0.9984) were achieved for GFAP, h-FABP and S100β, respectively.

### 3.3. Standard Recovery Test

The applicability and reliability of developed mTBI biomarker assays, on both ECL instrument platforms, were evaluated in a complex physiological matrix by standard addition method. Different concentrations of biomarkers were added in human serum diluted 2× with respective assay diluents (defined as “spiked” concentration). Based on the obtained ECL signal intensity, the corresponding biomarker concentrations (defined as “detected” concentration) were calculated according to the regression equation performed using the same matrix (4PL dose-response curve). The recoveries were calculated by the ratio between the “detected” and “spiked”, and the obtained results are summarized in Table 5 (Figure S5). It could be seen that the recoveries ranged from 79% to 128%, which seems acceptable at this development stage. These results suggest that with further development and optimization, these assays have a potential for future mTBI clinical diagnostic applications.

### 3.4. Multiplex ECL Assay for mTBI Biomarker Panel on SPCE

Once the *singleplex* assays have been developed on SPCE (GFAP, h-FABP, S100β) they were integrated into a multiplexed assay with the goal to enable high-throughput simultaneous detection of multiple mTBI biomarkers on a single electrode.

**Figure 5:**
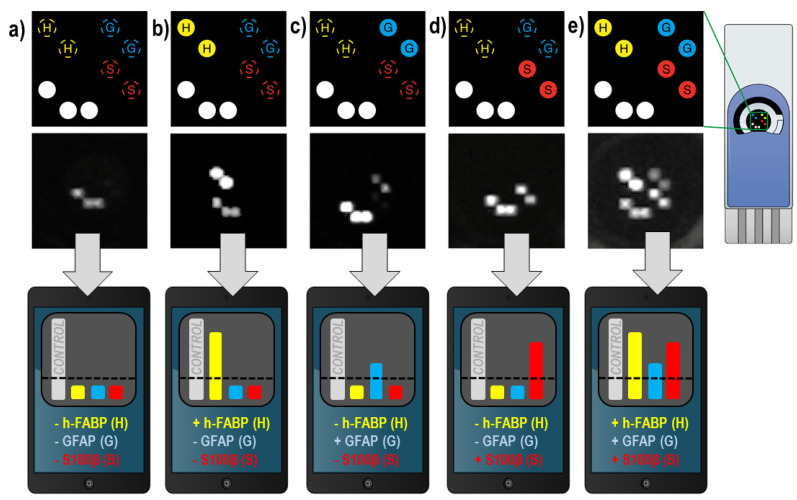
Multiplex ECL detection of mTBI biomarker panel established on a single SPCE. Top panel figures indicate the electrode patterns that contain alignment spots (“control”) made with BSA protein labelled with SULFO-TAG (white spots); spots for GFAP biomarker (blue spots with letter “G” indicate the position of GFAP capture antibodies); spots for h-FABP biomarker (yellow spots with letter “H” indicate the position of h-FABP capture antibodies); and spots for S100β biomarker (red spots with letter “S” indicate the position of S100β capture antibodies). Dashed letter spots indicate that no antigen was added (blank), while full-colored letter spots indicate that respective antigen was added in the assay. Middle panel figures respectively show real ECL images obtained from the electrode pattern indicated on top panel images, and bottom panel figures show the concept of the signal read-out displayed for the future envisioned mTBI POC diagnostic device: (**a**) biomarkers not detected in the sample (only three “control” spots are visible); (**b**) h-FABP biomarker detected; (**c**) GFAP biomarker detected; (**d**) S100β biomarker detected; (**e**) all three biomarkers detected.

SPCEs were spotted with capture antibodies of each biomarker (14 nL/spot, diameter ~500 µm) and with BSA protein labelled with SULFOTAG (alignment spots on the electrodes) (Figure 5), and the assay protocol was performed as described in Section 3.3 (S100β diluents were used as common diluents for all three biomarkers). Different electrode patterns were prepared, as described in Figure 5a–e. Obtained results indicated no cross-reactivity between the antibodies, providing a proof-of-concept that the present methodology based on spatially resolved approach can serve as a foundation for simultaneous, single electrode, detection of a mTBI biomarker panel, and other multi-biomarker panels (e.g., cardiac).

## 4. Discussion

The purpose of this study was to evaluate the feasibility of developing a multiplex ECL assay for mTBI biomarkers targeted for a POC diagnostic format and to identify the most important steps that need to be conquered on the way.

Firstly, ECL assays for three mTBI biomarkers (GFAP, h-FABP, S100β) have been developed on a reference benchtop ECL platform from MesoScale Discovery (96-well plate format). The analytical performances of these *singleplex* assays were impressive, reaching LODs of 6.94 pg mL^−1^, 1.35 pg mL^−1^ and 15.73 pg mL^−1^ for GFAP, h-FABP and S100β biomarker, respectively. The obtained LODs were close or below 1/10 of the cut-off values reported for these three biomarkers (22 pg mL^−1^, 2.62 ng mL^−1^ and 42 pg mL^−1^, Table 1), indicating that the developed assays have analytical sensitivities to distinguish a “mTBI condition” from “normal”, physiological concentration of the specific biomarker.

When comparing the obtained results with other published ECL-based methodologies, it is worth mentioning the work of Button et al., who reported a sandwich immunoassay for detection of GFAP biomarker on MSD platform in mouse plasma samples, with a LOD of 9 pg mL^−1^ [54]. Roche Diagnostics provides ECL-based immunoassay for the in vitro diagnostic quantitative determination of S100β in human serum based on Elecsys^®^ technology on Cobas instruments, with reported LOD of 15 pg mL^−1^ [55]. Gan et al. reported an ECL immunosensor for detection of h-FABP biomarker using luminophore coupled with 2D metal-organic framework (LOD of 44.5 fg mL^−1^) [31]. Regarding the methodologies reported for detection of mTBI biomarkers suitable for POC settings, it is worth mentioning the lateral flow immunoassay (LFIA) approaches reported by Natarajan and Joseph [56] for rapid detection of GFAP biomarker (time-resolved fluorescence read-out, 25 min detection time, LOD 10 pg mL^−1^), the approach of Savin et al. [57] for detection of h-FABP biomarker (CdTe quantum dots labelled detection antibodies, LOD 221 pg mL^−1^), and the approach developed by Gao et al. [58] for detection of S100β (surface-enhanced Raman spectroscopy read-out, LOD 5 pg mL^−1^). A detailed list of other methodologies published so far in the context of detection of GFAP, h-FABP and S100β biomarkers is given in Table S1.

Even though the MSD platform allows facile assay development and optimization, the instrument is not suitable for POC diagnostics and particularly not for on-site accident applications due to the size, weight, and cost limitations. Thus, the ECL assays for mTBI biomarkers have been translated to screen-printed electrodes (SPEs) coupled with a (trans-)portable µSTAT-ECL reader from Metrohm DropSens (see Section 2.1). SPEs are an attractive choice for realizing POC-oriented devices due to their low cost, mass fabrication, small sample volume requirements, and ability to be easily integrated and miniaturized [59]. SPEs can be easily combined with ECL detection to achieve a cost-effective, simplified POC diagnostic device.

One of the most important electrode aspects in the context of ECL detection is the electrode material. Our results have shown that non-modified SPCE exhibit better performances in the ECL assays than all other tested electrodes from Metrohm DropSens (SPCE-GNP, SPCE-CNT-GNP, or SPCE-QD). A similar observation was also reported (excluding SPCE-QD) in the work of Kerr et al. [49]. Unmodified carbon is often chosen for WE since it is a cheap, widely commercially available material that exhibits rapid and efficient oxidation of TPA, is relatively hydrophobic, allowing high concentrations of TPA on the electrode surface, and has low rates of surface oxide formation compared to noble metal electrodes [49,52]. Furthermore, two different electrochemical techniques were employed for ECL signal trigger, linear sweep voltammetry (LSV) and constant potential chronoamperometry (CPA). The obtained results showed that CPA at 1.55 V increased ~3× the ECL signal intensities in the assay compared to the LSV (Figure 3b).

Even though in the literature there are plenty of ECL biosensors based on screen-printed electrodes [60], to the best of our knowledge, this is the first study showing the development of ECL assays on SPCE combined with the miniaturized µSTAT-ECL reader from Metrohm DropSens [41]. The LOD values of 0.59, 0.44, and 1.34 ng mL^−1^ were achieved for GFAP, h-FABP and S100β biomarker, respectively. The evident loss of sensitivity (compared to the MSD platform) could likely be attributed to the less performant μSTAT-ECL photodiode versus MSD CCD detector systems. On the other hand, new, highly sensitive and compact detectors in development will likely soon unfold possibilities for low pg mL^−1^ level LODs in POC diagnostic assays. Alternatively, for SPCEs to be applicable for detection of a mTBI-relevant biomarker panel at the clinically relevant concentration ranges (lower pg mL^−1^ range), further developments with ECL signal amplification strategies (e.g., exploring nanomaterials that have a higher capacity for loading of luminophores—e.g., solid-state luminophores) would be highly beneficial.

In terms of the simultaneous detection of multiple analytes, a variety of analytical technologies have been exploited, including fluorescence [61,62], electrochemistry [63,64], surface plasmon resonance [65], and chemiluminescence [66]. However, some techniques need expensive detectors or light sources, while at the same time may suffer insufficient sensitivity for measuring clinical samples. Electrochemiluminescence has shown an excellent potential thanks to the good selectivity and the fact that signal generation can be adjusted by the alternation of the trigger potential, allowing control over emission location and thus enabling multi-analyte detection from the sample, by employing single or multiple electrodes [67]. The methodologies for ECL-based multiplex assays can be mainly categorized as spatial-resolved, potential-resolved, spectrum-resolved, and other miscellaneous strategies [67]. Herein the spatially resolved approach on a single SPCE has been applied for the first time for multiplex ECL detection of three mTBI biomarkers. Translation of three individual *singleplex* assays into multiplex assay has been successfully done, indicating that such an approach could be further explored for detection of mTBI biomarker panel from limited sample volume (e.g., capillary blood sample, cerebrospinal fluid, etc.), and with the option to extend the biomarker panel and improve the clinical specificity of the test (e.g., fewer false positives).

Due to the differences in mTBI biomarker release kinetics, a multi-point detection at different time intervals after the injury would be an important requirement. For example, GFAP is present in serum samples at detectable concentrations (>30 pg mL^−1^) even within 1 h of injury [68], while for S100β only 12–36 h after trauma. The methodology could certainly benefit both clinicians and patients by being easily extended to other multi-target panels (cancer, cardiac disease, etc.). The spatially resolved approach evidently brings the requirement for the use of a CCD camera and for POC diagnostic applications, it would be necessary to employ a miniaturized and performant detector device and a cartridge unit that would handle all the sample processing steps.

## 5. Conclusions

Herein, electrochemiluminescence assays for detection of mTBI biomarkers (GFAP, h-FABP, S100β) have been developed and optimized on the MesoScale Discovery (MSD) platform in 96-well plate format, and on SPCE electrodes combined with µSTAT-ECL from Metrohm DropSens. A “proof-of-concept” for the development of a multiplex ECL assay on a single electrode has been shown. Obtained data could serve as a steppingstone towards the development of an ECL-based POC diagnostic device for multiplex detection of mTBI biomarkers, which can be employed as a diagnostic screening test, but also aid the preclinical evaluation of currently investigated biomarkers and in the establishment of an ‘ideal’ biomarker panel for TBI diagnostics (or adapted for the detection of other biomarker panels in different clinical body fluids).

## Figures and Tables

**Table 1:** mTBI protein biomarkers used in this work and their clinically relevant concentration ranges.

Biomarker		Physiological Concentration ^1^	
Abbreviation	Full Name	Normal	Mild TBI
GFAP	Glial fibrillary acidic protein	RG: 2–49 pg mL^−1^ [18] MD: 4 pg mL^−1^ [18]	≥33 pg mL^−1^ [18] CO: 22 pg mL^−1^ [27]
h-FABP	Heart-fatty acidic binding protein	<5.5 ng mL^−1^ [34] MN: 3.78 ng mL^−1^ [35]	CO: 2.62 ng mL^−1^ (HS/HP) [23]
S100β	S100β calcium-binding protein	MD: 50 pg mL^−1^ (HP) [36] <0.11 pg mL^−1^ [37]	≥100 pg mL^−1^ (HP) [38] >75 pg mL^−1^ [39] CO: 42 pg mL^−1^ (HS/HP) [23]

^1^ Physiological concentration is indicated for human serum unless otherwise specified. Values reported in samples other than blood/serum/plasma (e.g., sweat, urine, muscle-on-tissue, etc.) are not considered. Abbreviations: CO—cutoff value; HP—human plasma; HS—human serum; MN—mean value; MD—median value; RG—range.

**Table 2:** Preliminary conditions evaluated on MSD platform for each individual mTBI biomarker ECL sandwich immunoassay (*singleplex*).

mTBI Biomarker	cAb Diluent	Blocking Agent	Assay Diluent	Wash Buffer	dAb Diluent	cAb Conc. (μg mL^−1^)	dAb Conc. (μg mL^−1^)	LOD (pg mL^−1^)	Range (pg mL^−1^)
GFAP	PBS 1×	1% BSA; 0.06% Tween-20; PBS 1×	MQ water	0.06% Tween-20; PBS 1×	1% BSA; PBS 1×	25.00	2.50	6.94 [6.9–10.0; CI:95%]	0–10,000
h-FABP	PBS 1×	1% BSA; 0.06% Tween-20; PBS 1×	PBS 1×	0.06% Tween-20; PBS 1×	1% BSA; PBS 1×	5.00	0.50	1.35 [0–4.4; CI:95%]	0–10,000
S100β	5 mM CaCl_2_; 50 mM TRIS	2% BSA; 1 mM CaCl_2_; PBS 1×	0.1% BSA; 1 mM CaCl_2_; 50 mM TRIS	0.06% Tween-20; PBS 1×	0.1% BSA; 1 mM CaCl_2_; 50 mM TRIS	10.00	5.00	15.73 [11.0–20.5; CI:95%]	0–10,000

**Table 3:** Electrochemical properties of tested commercially available SPEs.

SPE	Product Number	A (cm^2^) *	ΔE (mV)	I_a_/I_c_
SPCE	DRP-110	0.1540	150	1.04
SPCE-GNP	DRP-110-GNP	0.1580	180	1.00
SPCE-CNT-GNP	DRP-110CNT-GNP	0.1470	285	1.03
SPCE-QD	DRP-110QD	0.0270	270	1.00

* Calculated using Equation (2), (average n = 3).

**Table 4:** Preliminary conditions evaluated on SPCE using µSTAT-ECL platform for each individual mTBI biomarker ECL sandwich immunoassay (*singleplex*).

mTBI Biomarker	cAb Diluent	Blocking Agent	Assay Diluent	Wash Buffer	dAb Diluent	cAb Concentration (μg mL^−1^)	dAb Concentration (μg mL^−1^)	LOD	Dynamic Range
GFAP	PBS 1×	1% BSA; 0.06% Tween-20; PBS 1×	MQ water	0.06% Tween-20; PBS 1×	1% BSA; PBS 1×	25.00	2.50	0.59 ng mL^−1^	0–50 ng mL^−1^
h-FABP	PBS 1×	1% BSA; 0.06% Tween-20; PBS 1×	PBS 1×	0.06% Tween-20; PBS 1×	1% BSA; PBS 1×	5.00	0.50	0.44 ng mL^−1^	0–50 ng mL^−1^
S100β	5 mM CaCl_2_; 50 mM TRIS	2% BSA; 1 mM CaCl_2_; PBS 1×	1 mM CaCl_2_; 50 mM TRIS	0.06% Tween-20; PBS 1×	0.1% BSA; 1 mM CaCl_2_; 50 mM TRIS	25.00	12.50	1.34 ng mL^−1^	0–50 ng mL^−1^

**Table 5:** Results of the recovery test for mTBI biomarkers in human serum (HS, n = 2) samples diluted 2× with respective assay diluent (details of the assay conditions are listed in Table 2).

Sample	Added Biomarker	Spiked Concentration (ng mL^−1^)	Detected Concentration by MSD (ng mL^−1^)	Recovery (%)	Detected Concentration by µSTAT-ECL (ng mL^−1^)	Recovery (%)
Human serum	h-FABP	0	0	/	0	/
		2.50	2.30	92%	1.99	79%
		5.00	4.37	87%	5.33	106%
	GFAP	0	0	/	0	/
		2.50	2.23	89%	2.62	105%
		5.00	5.30	106%	4.34	87%
	S100β	0	0	/	0	/
		2.50	2.43	96%	2.11	84%
		5.00	6.41	128%	5.92	118%

## Data Availability

The experimental data is contained within the article.

## Supplementary Materials

The following supporting information are available online at https://www.mdpi.com/article/10.3390/bios12030172/s1, Table S1: Summary of GFAP, h-FABP and S100β sensors developed in the last 10 years and their analytical performances; Figure S1: Four-parameter (4PL) dose-response nonlinear regression model for MSD calibration curves; Figure S2: Electrochemical characterization of commercially available screen-printed electrodes (SPE) for ECL applications; Figure S3: SEM images of commercially available Screen-Printed Cabon Electrodes (SPCEs) for ECL applications; Figure S4: Linear regression model for SPCE µSTAT-ECL calibration curves; Figure S5: Results of the recovery studies; Figure S6: CAD drawing of incubation cells used for SPEs. References [27,30,31,32,54,55,56,57,58,69,70,71,72,73,74,75,76,77,78,79,80,81,82,83,84,85,86,87,88,89,90,91,92,93,94,95,96,97,98,99,100,101,102,103,104,105,106,107,108,109,110,111,112,113,114,115,116,117,118,119] are cited in the supplementary materials.
