# Supplementary material for: Towards a Point-of-Care (POC) Diagnostic Platform for the Multiplex Electrochemiluminescent (ECL) Sensing of Mild Traumatic Brain Injury (mTBI) Biomarkers
Source: Biosensors (Basel). 2022 Mar 11;12(3):172. doi: 10.3390/bios12030172 (PMC8946848; doi:10.3390/bios12030172)
Supplement: Supplementary file 1 [file biosensors-12-00172-s001.zip › biosensors-1601928-supplementary.pdf]

Supplementary Information

Towards a Point-of-Care (POC) Diagnostic Platform for the Multiplex Electrochemiluminescent (ECL) Sensing of Mild Traumatic Brain Injury (mTBI) Biomarkers

M. Jović, D. Prim, E. Saini, M. E. Pfeifer*

[1] University of Applied Sciences and Arts Western Switzerland (HES-SO Valais-Wallis), Institute of Life Technologies, Diagnostic Systems Research Group, rue de l'Industrie 23, 1950 Sion, Switzerland; info.itv@hevs.ch

* Correspondence: marc.pfeifer@hevs.ch; Tel.: +41 58 606 86 61

Table S1: Summary of GFAP, h-FABP and S100β sensors developed in the last 10 years and their analytical performances. 2

Figure S1: Four-parameter (4PL) dose-response nonlinear regression model for MSD calibration curves. 12

GFAP 12

h-FABP 13

S100β 14

Figure S2: Electrochemical characterization of commercially available screen-printed electrodes (SPE) for ECL applications. 15

Figure S3: SEM images of commercially available Screen-Printed Carbon Electrodes (SPCEs) for ECL applications. 17

Figure S4: Linear regression model for SPCE µSTAT-ECL calibration curves. 18

GFAP 18

h-FABP 19

S100β 20

Figure S5: Results of the recovery studies. 21

Figure S6: CAD drawing of incubation cells used for SPEs. 22

References 23

Table S1: Summary of GFAP, h-FABP and S100β detection strategies reported in the literature and commercialized products and assay kits.

Biomarker	Brief description of method/ Detection approach	Sensor/Assay architecture	Publication year and reference	Lower Detection Limit (1)	Range (2)	Tentative suitability for POC applications (*advantages/ disadvantages*) (3)
			- Academic publications -			
GFAP *Glial fibrillary acidic protein* *(1/3)*	Label-free biosensor Electrochemical impedance spectroscopy (EIS) read-out	Au MDEA or Au MECS/TSP/Ab/T	2013 [1]	1 pg mL^{-1} (buffer)	1 pg mL^{-1}–100 ng mL^{-1}	✓ Label free<br>✓ Sample volume (60 μL)<br>X Complex fabrication
	Organic thin film transistor (TFT) protein sensor FED (OFET)	Si/SiO_2/(w/wo Pentacene or 8-3 NTCDI)/CYTOP/C44H90/ NHS-PS-block PAA (EDC+NHS)/Ab/T	2014 [2]	1 ng mL^{-1} (buffer)	0.8-400 ng mL^{-1}	✓ Label free<br>X Sensitivity<br>X Complex fabrication
	Molecularly Imprinted Polymer (MIP) sensor Electrochemical read-out (DPV)	MIP-MWCNTs: (MWCNTs+AIBN+ DMAA+AEDP+ EGDMA[T])/agarose film/(SDS+HCl)/EDTA	2017 [3]	0.04 μg mL^{-1} (buffer)<br><0.9 μg mL^{-1} (HS)	0.2-10 μg mL^{-1}	✓ Label free<br>✓ Sample volume (50 μL)<br>X Sensitivity<br>X Complex fabrication
	Organic field effect transistor biosensor (OFET)	Drive: Si/SiO_2/Pentacene/Au Sensing: Si/(PS-MA+PEG)/ Ab/T	2017 [4]	1 ng mL^{-1} (buffer)	0.5-100 ng mL^{-1}	✓ Label free<br>X Sensitivity
	Label-free biosensor Electrochemical impedance spectroscopy (EIS) read-out	Graphene SPE/NaOH (-OH)/PEI/GA/Ab/T	2018 [5]	1 pg mL^{-1} (buffer)<br>1 pg mL^{-1} (HS)	1 pg mL^{-1}–100 ng mL^{-1}	✓ Label free<br>✓ Sensitivity<br>✓ Sample volume (50 μL)<br>✓ Detection in serum and CSF samples
	Immunosorbent assay with Carbon Dots (CD) Fluorescence read-out	Protein A/G/Ab_1/T/Ab_2/CDs	2018 [6]	25 pg ml^{-1}	0.1–8 ng ml^{-1}	✓ Sensitivity<br>✓ Detection in serum samples<br>✓ Potential for Multiplexing

Biomarker	Brief description of method/ Detection approach	Sensor/Assay architecture	Publication year and reference	Lower Detection Limit (1)	Range (2)	Tentative suitability for POC applications (*advantages/ disadvantages*) (3)
GFAP *Glial fibrillary acidic protein* *(2/3)*	Label-free biosensor Single frequency impedance electrochemical spectroscopy (EIS SFI) read-out	Au DE/MHDA/(EDC+NHS)/Ab/T	2019 [7]	2-5 pg mL^{-1} 14-67 pg mL^{-1} (90% whole blood)	0.1-2800 pg mL^{-1}	✓ Label free ✓ Sensitivity ✓ Multiplexing (4 biomarkers) ✓ Detection in blood samples
	QCM sensors Ultra-high frequency surface acoustic wave (SAW) read-out	F1: Au/PEG/streptavidin/biotin-Ab/T F2: Au/Protein G/Ab/T F3: Au/S-S/Ab/T	2021 [8]	35 pM (buffer and serum)	n/a	✓ Lab-on-a-chip ✓ Detection in serum samples
	Sandwich Immunoassay Electrochemiluminescence read-out (ECL) on MesoScale Discovery platform	SPCE/Ab_1/T/Ab_2-Ru label	2021 [9]	9 pg mL^{-1} (buffer and plasma)	200 pg ml^{-1}-200 ng ml^{-1}	✓ Sensitivity X Currently developed for benchtop instrument
	Magnetic bead (MB)-based quantum dot-linked immunosorbent assays (QLISAs) in variable height microfluidic device Fluorescence read-out	MB-Ab_1/T/Ab_2-QD	2021 [10]	125 pg mL^{-1} (serum) 1112 pg mL^{-1} (whole blood)	0.1-10 ng ml^{-1}	X Sensitivity ✓ Detection in serum/blood samples ✓ Multiplexing (3 biomarkers) ✓ POC format
	Lateral flow immunoassay (LFIA) Time-resolved fluorescence (TRF) read-out	Ab_1/T/Ab_2-CM EUs	2022 [11]	10 pg mL^{-1} (whole blood)	0-400 pg mL^{-1}	✓ Sensitivity ✓ POC format ✓ Detection in blood samples ✓ 25 min analysis time
	- Commercial products (RUO/diagnostic instruments, devices, or kits) -					
	ELISA kit (Sigma Aldrich) Colorimetric read-out *Research use only*	Microtiter plate/Ab_1/T/Ab_2-biotin/streptavidin HRP	GFAP ELISA Kit, NS830-M [12]	n/a	1.5-100 ng ml^{-1}	Intended for benchtop platforms

Biomarker	Brief description of method/ Detection approach	Sensor/Assay architecture	Publication year and reference	Lower Detection Limit (1)	Range (2)	Tentative suitability for POC applications (*advantages/ disadvantages*) (3)
GFAP *Glial fibrillary acidic protein* *(3/3)*	Sandwich immunoassay kit Electrochemiluminescence (ECL) read-out (MesoScale Discovery) *Research use only*	Microtiter plate with SPCE/Ab_1/T/Ab_2-SULFO TAG label	R-PLEX Human GFAP Antibody Set (F211M-3/-8) [13]	63 pg mL^{-1}	≤ 500 ng ml^{-1}	Intended for MesoScale Discovery instruments
	ELISA kit with chemiluminescence (CL) read-out Banyan BTI™ *In vitro diagnostic kit*	Microtiter plate/Ab_1/T/Ab_2/substrate	Banyan BTI™ [14,15]	10 pg mL^{-1} (human serum)	10-320 pg mL^{-1}	Intended for benchtop platforms
	ELISA kit (Quanterix Simoa®) Fluorescence read-out *In vitro diagnostic kit* *(*License from Banyan Biomarkers)*	MB-Ab_1/T/Ab_2-label	GFAP Discovery kit* [16]	0.467 pg mL^{-1} (human CSF, serum and plasma)	0-4'000 pg mL^{-1}	Intended for Quanterix Simoa instruments (SR-X, HD-1, HD-X)
	Hand-held device i-STAT Alinity Abbott Point of Care Inc Amperometric read-out *In vitro diagnostic device* *(*License from Banyan Biomarkers)*	Au electrode/(MB-)Ab_1/T/Ab_2-ALP	Duplex (GFAP & UCH-L1)* cartridge [17–20]	23 pg mL^{-1} (human plasma)	30-10'000 pg mL^{-1}	Intended for POC
	Metal-oxide semi-conducive (CMOS) compatible nanosensors (NanoDx)	Nanowires/Ab/T	TBI Duplex (GFAP & S100 β) [21]	n/a	n/a	Intended for POC *Prototype under development*

Biomarker	Brief description of method/ Detection approach	Sensor/Assay architecture	Publication year and reference	Lower Detection Limit (1)	Range (2)	Tentative suitability for POC applications (*advantages*/ *disadvantages*) (3)
			- Academic publications -			
h-FABP *Heart-fatty acidic binding protein* *(1/3)*	Sandwich immunoassay Square-wave voltammetry (SWV) read-out	GCE/GRONRs/(EDC+NHS)/ Ab_1/T/Ab_2/GA/$TiPZn^{2+}$-probe	2012 [22]	3 fg mL^{-1} (buffer) <1.7 μg mL^{-1} (HS)	0.05 pg mL^{-1} –50 ng mL^{-1}	✓ Sensitivity<br>✓ Multiplexing<br>✓ Sample volume (20 μL)<br>✓ Detection in serum samples
	Label-free biosensor Electrochemical impedance spectroscopy (EIS) read-out	Au/ MUA/(EDC+NHS)/Ab/T [mSAM] Au/(MUA+MPOH)/(EDC+NHS)/Ab/ T [hSAM]	2012 [23]	117 pg mL^{-1} *[mSAM]*; 524 pg mL^{-1} *[hSAM]* (buffer)	98 pg mL^{-1}-100 ng mL^{-1}	✓ Label free<br>X Sensitivity<br>X Complex fabrication
	Immunoassay Turbidimetric read-out	Latex particles/T	2013 [24]	2.4 ng mL^{-1}	2.76 ng mL^{-1}-115 ng mL^{-1}	X Sensitivity
	Label-free capacitive interdigitated immunosensor	AuIDEs/ MUA/(EDC+NHS)/Ab/T [mSAM] AuIDEs/(MUA+MPOH)/(EDC+ NHS)/Ab/T [hSAM]	2015 [25]	0.836 ng mL^{-1} *[mSAM]* 0.968 ng mL^{-1}*[hSAM]* (buffer)	98 pg mL^{-1}–100 ng mL^{-1}	✓ Label free<br>✓ Sample volume (50 μL)<br>X Sensitivity<br>X Complex fabrication
	Sandwich immunoassay Anodic stripping voltammetry (ASV) read-out	GCE/CD-GS/Ab_1/T/Ab_2-ZnO-MWCNTs/CdS	2017 [26]	0.3 fg mL^{-1} (buffer) <5 pg mL^{-1} (HS)	1.3 fg mL^{-1}-130 ng mL^{-1}	✓ Sensitivity<br>✓ Multiplexing<br>✓ Sample volume (6 μL)<br>✓ Detection in serum samples
	Label-free immunosensor Electrochemiluminescence (ECL) read-out	GCE/MOF-Ru/GA-CS/Ab/T	2018 [27]	2.6 fg mL^{-1}	150 fg mL^{-1}-150 ng mL^{-1}	✓ Label-free<br>✓ Sensitivity<br>X Complex fabrication
	Lateral flow immunoassay (LFIA) with quantum dots (QD) Fluorescence read-out	Ab_1/T/Ab_2-CdTe	2018 [28]	221 pg mL^{-1}	0-160 ng mL^{-1}	X Sensitivity<br>✓ POC format<br>✓ Detection in blood samples

Biomarker	Brief description of method/ Detection approach	Sensor/Assay architecture	Publication year and reference	Lower Detection Limit (1)	Range (2)	Tentative suitability for POC applications (*advantages*/ *disadvantages*) (3)
h-FABP<br>*Heart-fatty acidic binding protein*<br><br>*(2/3)*	Molecularly imprinted polymer nanoparticles (nanoMIPs) biosensor<br>Thermal read-out	Thermocouple/MIP/T	2019 [29]	1.5 ng mL^{-1}	1.5 ng mL^{-1}-75 ng mL^{-1}	X Sensitivity<br>✓ POC format<br>✓ Multiplexing
	Sandwich immunoassay<br>Paper based (µPAD) device<br>Chemiluminescence (CL) read-out	GA-CS/Ab1-GNP/T/Co(II)-luminol-GNP	2020 [30]	0.06 pg mL^{-1}	0.1 pg mL^{-1}-1 µg mL^{-1}	✓ Sensitivity<br>✓ POC format<br>✓ Multiplexing
	Immunosensor with luminophore coupled with 2D MOF<br>Electrochemiluminescence (ECL) read-out	GCE/PICA/Ab_1/T/Ab_2-Ni-TCPP(Fe)-PEI-luminol	2021 [31]	44.5 fg mL^{-1}	100 fg mL^{-1}-100 ng mL^{-1}	✓ Sensitivity<br>X Complex fabrication<br>✓ Detection in serum samples
	- Commercial products (RUO/diagnostic instruments, devices, or kits) -					
	Lateral flow immunoassay (LFIA)<br>(TBI Check ABCDx)<br>Colorimetric read-out<br>*In vitro diagnostic test*	Ab_1/T/Ab_2-label	TBI Check ABCDx [32]	n/a	n/a	Intended for POC
	ELISA kit (Abcam)<br>Colorimetric read-out<br>*Research use only*	Microtiter plate/Ab_1/T/Ab_2-biotin/streptavidin HRP	Human H-FABP ELISA Kit (ab243682) [33]	n/a	10.9-700 pg mL^{-1}	Intended for benchtop platforms
	ELISA kit (Cusabio)<br>Colorimetric read-out<br>*Research use only*	Microtiter plate/Ab_1/T/Ab_2-biotin/streptavidin HRP	ELISA Kit (CSB-E09185h)	n/a	78-5000 pg mL^{-1}	Intended for benchtop platforms

Biomarker	Brief description of method/ Detection approach	Sensor/Assay architecture	Publication year and reference	Lower Detection Limit (1)	Range (2)	Tentative suitability for POC applications (*advantages/ disadvantages*) (3)
h-FABP *Heart-fatty acidic binding protein* *(3/3*	Sandwich immunoassay kit Electrochemiluminescence (ECL) read-out (MesoScale Discovery (MSD)) *Research use only*	licrotiter plate with SPCE/Ab_1/T/Ab_2-SULFO TAG label	Human FABP3/H-FABP Antibody Set MesoScale Discovery (MSD) (F214T) [34]	90 pg mL^{-1}	≤ 100 ng ml^{-1}	Intended for MesoScale Discovery instruments
	- Academic publications -					
S100β *S100β calcium-binding protein* *(1/5)*	Sandwich immunoassay Fluorescence read-out	Microtiter plate/Ab_1/T/Ab_2-biotin/streptavidin europium	1997 [35]	15 pg mL^{-1} (plasma)	n/a	✓ Sensitivity<br>X Currently developed for microtiter plate format<br>✓ Detection in plasma samples
	ELISA Colorimetric read-out	Microtiter plate/Ab_1/T/Ab_2-HRP	1997 [36]	40 pg mL^{-1} (CSF)	0.5-2.5 ng mL^{-1}	X Currently developed for microtiter plate format<br>✓ Detection in CSF samples
	ELISA Colorimetric read-out	Microtiter plate/ Ab_1/T/Ab_2/antiAb_2-HRP	2008 [37]	n/a	1.9 pg mL^{-1}-10 ng mL^{-1} (human and rat samples)	✓ Sensitivity<br>X Currently developed for microtiter plate format<br>✓ Detection in real samples
	Sandwich immunoassay Differential pulse voltammetry (DPV) read-out	Pencil graphite/PMMA/-OH(NaOH)/NH_2(PEI)/GA/Ab_1/T /Ab_2/ALP-IgG	2013 [38]	0.1 pg mL^{-1} (buffer)	0.1-100 pg mL^{-1}	✓ Sensitivity<br>✓ Sample volume (20 μL)<br>X Complex fabrication
	Sandwich immunoassay Square-wave voltammetry (SWV) read-out	Au DE/(capture peptide+TCEP)/ (T+$CaCl_2$)/(signal peptide+Cu^{2+})	2014 [39]	0.1 nM (buffer) <0.2 nM (HS)	0.1-25.6 nM	X Complex fabrication<br>✓ Detection in serum samples

Biomarker	Brief description of method/ Detection approach	Sensor/Assay architecture	Publication year and reference	Lower Detection Limit (1)	Range (2)	Tentative suitability for POC applications (*advantages/ disadvantages*) (3)
S100β<br>*S100β calcium-binding protein*<br>*(2/5)*	Label-free biosensor with His-RAGE domains, Osteryoung square-wave voltammetry (OSWV) read-out	Au DE/(DPTA+NAC)/Cu^{2+}/His_6-RAGE VC1 or C2/T	2014 [40]	0.52 pM (buffer)<br>0.65 pM (HP)	1-20 pM	✓ Label-free<br>✓ Sample volume (10 μL)<br>X Complex fabrication<br>✓ Detection in plasma samples
	Magnetic bead (MB) based quantum dot (QD) immunoassay<br>Fluorescence read-out	MB-Ab_1/T/Ab_2-QD	2015 [41]	10 pg mL	0.01–30 ng mL^{-1}	✓ Sensitivity
	Label-free biosensor with His-RAGE domains, Osteryoung square-wave voltammetry (OSWV) read-out	Au DE/(DPM+NAC)/Cu^{2+}/His_6-RAGE VC1 or C2/T<br>(a) (DPM+MBT)/Cu^{2+}/His_6-RAGE VC1 or C2/T (b)	2016 [42]	2.6 pM (a) (buffer)<br>4.9 pM (b) (buffer)<br>0.9 pM (a) (HP)<br>2.7 pM (b) (HP)	2.6-20 pM (a)<br>4.9-20 pM (b)<br>0.9-20 pM (a)<br>2.7-20 pM (b)	✓ Label free<br>✓ Sample volume (10 μL)<br>X Complex fabrication<br>✓ Detection in plasma samples
	Label-free biosensor<br>Differential pulse voltammetry (DPV) read-out	Electrografted reduced FRGG/GA/Ab/T	2017 [43]	1 pg mL^{-1} (buffer)<br>1 pg mL^{-1} (HS and CSF)	1 pg mL^{-1}-10 ng mL^{-1} (buffer)<br>1 pg mL^{-1}-10 ng mL^{-1} (HS and CSF)	✓ Label free<br>✓ Sensitivity<br>✓ Detection in serum and CSF samples
	Label-free biosensors<br>Electrochemical impedance spectroscopy (EIS) read-out	Au IDE/(4-ATP + cysteamine)/ GA/Ab/T	2018 [44]	10 ng mL^{-1} (buffer)	10 ng mL^{-1}-10 μg mL^{-1}<br>*(vs lgC$_T$)*	✓ Label free<br>X Sensitivity<br>✓ Multiplexing
	Surface-enhanced Raman scattering (SERS) biosensor	ITO/HAuNPs/DMSA + EDC-NHS/Ab_1/HAuNPs/4-MBA(NBA)/EDC-NHS/Ab_2	2018 [45]	0.06 ng mL^{-1}	0.2-22 ng mL^{-1}	✓ Sensitivity<br>✓ Multiplexing<br>X Complex fabrication
	Sandwich immunoassay<br>Field-effect enzymatic detection (FEED)	Carbon SPE/SWCNTs-Nafion-GA/Ab_1/T/HRP-Ab_2	2018 [46]	10 fg mL^{-1} (HS)	10 fg mL^{-1}-10 ng mL^{-1}	✓ Reagent-less<br>✓ Sensitivity<br>✓ Detection in serum samples

Biomarker	Brief description of method/ Detection approach	Sensor/Assay architecture	Publication year and reference	Lower Detection Limit (1)	Range (2)	Tentative suitability for POC applications (*advantages/ disadvantages*) (3)
S100β *S100β calcium-binding protein* *(3/5)*	Lateral flow immunoassay (LFIA) Square-wave voltrammetry (SWV) or SERS read-out	FTO/AgNPs/Au/4-MBA/Ab/T	2019 [47]	10 pg mL^{-1} (buffer) <1.8 ng mL^{-1} (HS)	50 pg mL^{-1}-1 μg mL^{-1}	✓ Label free<br>✓ Sensitivity<br>✓ Multiplexing<br>✓ Detection in serum samples
	Sandwich immunoassay Photo-electrochemical (PEC) read-out	ITO/rGRO-AuNPs/3-ICT-sol-gel-film/Ab_1/T/Ab_2/(EDC+NHS)/CdS-QDs	2019 [48]	0.15 pg mL^{-1} (buffer) <100 pg mL^{-1} (HS)	0.25-10000 pg mL^{-1}	✓ Sensitivity<br>✓ Detection in serum samples<br>X Complex fabrication
	Surface plasmon resonance (SPR) biosensor	Point-of-care SPR module (PhotonicSys SPR H5) Au sensor/MUA/EDC-NHS/Ab/T	2019 [49]	0.75 ng mL^{-1} (water) 0.136 ng mL^{-1} (plasma)	0.25–10 ng mL^{-1}	X Sensitivity<br>✓ Multiplexing<br>✓ POC format<br>✓ Detection in plasma samples
	Biosensor with C/AuNCs nanoprobe Fluorescent read-out	C/AuNCs/TRTK-12 peptide/Ca^{2+}/T	2020 [50]	0.01 μg mL^{-1}	0.03-1 μg mL^{-1}	X Sensitivity
	Protein reduction/chemisorption on electrode Differential pulse voltammetry (DPV) read-out	Au DE Recognition probe: MBs/Au/Ab/T	2020 [51]	10 pM (buffer) <250 pM (horse plasma)	10 pM-100 nM	✓ Label free<br>✓ Sample volume (50 μL)<br>X Limited to analytes with disulphide bonds
	Lateral flow immunoassay (LFIA) SERS read-out	Ab_1/T/Ab_2-SERS probe	2021 [52]	5 pg mL^{-1} (HP)	5 pg mL^{-1}- 100 ng mL^{-1}	✓ Sensitivity<br>✓ Detection in plasma samples<br>✓ POC format

Biomarker	Brief description of method/ Detection approach	Sensor/Assay architecture	Publication year and reference	Lower Detection Limit (1)	Range (2)	Tentative suitability for POC applications (*advantages/ disadvantages*) (3)
			- Commercial products (RUO/diagnostic instruments, devices, or kits) -			
S100β<br>*S100β calcium-binding protein*<br>*(4/5)*	Sandwich immunoassay Electrochemiluminescence (ECL) read-out (Roche Diagnostics)<br>*In vitro diagnostic kit*	MB/Ab$_1$/T/Ab$_2$-ECL label	Elecsys® S100 test kit for human serum [53]	15 pg mL^{-1} (HS)	0.005-39 µg mL^{-1} (cobas e411, e601/ e602)<br>0.015-30 µg mL^{-1} (cobas e801)	Intended for Roche cobas instruments cobas e411, cobas e601, cobas e602; cobas e801
	Metal-oxide semi-conducive (CMOS) compatible nanosensors (NanoDx)	Nanowires/Ab/T	TBI Duplex (GFAP & S100 β) [21]	n/a	n/a	Intended for POC<br>*Prototype under development*
	CLIA kit (DiaSorin Liaison®) Chemiluminescence (CL) read-out<br>*In vitro diagnostic kit*	MB-Ab$_1$/T/Ab$_2$-label/substrate	S100 test kit [54]	20 pg mL^{-1} (HS)	n/a	Intended for Diasorin Liaison instruments
	ELISA kit (Fujirebio CanAg) Colorimetric read-out<br>*Research use only*	Microtiter plate/Ab$_1$/T/Ab$_2$-biotin/streptavidin HRP	S100 EIA [55]	10 pg mL^{-1} (HS)	0-3500 ng mL^{-1}	Intended for benchtop platforms
	ELISA kit (Sigma Aldrich) Colorimetric read-out<br>*Research use only*	Microtiter plate/Ab$_1$/T/Ab$_2$-biotin/streptavidin HRP	Human S100B, EZHS100B-33K [56]	n/a	2.7-2000 pg mL^{-1}	Intended for benchtop platforms
	ELISA kit (Abcam) Colorimetric read-out<br>*Research use only*	Microtiter plate/Ab$_1$/T/Ab$_2$-biotin/streptavidin HRP	S100B ELISA Kit (ab234573) [57]	n/a	0.31-20 ng mL^{-1}	Intended for benchtop platforms
	ELISA kit (Lucerna Chem) Colorimetric read-out<br>*Research use only*	Microtiter plate/Ab$_1$/T/Ab$_2$-biotin/streptavidin HRP	Human S100B [58]	n/a	93.75-6000 pg mL^{-1}	Intended for benchtop platforms

Biomarker	Brief description of method/ Detection approach	Sensor/Assay architecture	Publication year and reference	Lower Detection Limit (1)	Range (2)	Tentative suitability for POC applications (*advantages/ disadvantages*) (3)
S100β<br>*S100β calcium-binding protein*<br><br>*(5/5)*	ELISA kit (Genway Biotech)<br>Colorimetric read-out<br>*Research use only*	Microtiter plate/Ab_1/T/Ab_2-biotin/streptavidin HRP	S100b ELISA Kit (Rabbit) (GWB-KBBVZ1) [59]	n/a	15.6-1000 pg mL^{-1}	Intended for benchtop platforms

(1) Lowest reported LDL using EC detection methods; '<x' corresponds to the lowest concentration analyzed within the working range of the sensor (employing standard addition method and/or a reference material/method for validation, with a decent recovery), actual LDL being possibly lower than the indicated value. (2) The upper limit of the range indicated often presents the maximum concentration explored but not the upper detection limit. Please consult original paper for details. (3) Assessing the suitability for POC clinical diagnostic applications is difficult particularly if the described approaches are in early, e.g., proof-of-principle phase. For instance, many publications do no provide robustness, repeatability / reproducibility data (e.g., with real samples) nor have cost-of-goods produced (COGP), manufacturability and usability aspects (e.g., time to results) considered. A check sign (✓) in the table denotes a positive aspect observed, while a cross (X) either means a possibly problematic approach (for product development) or absence or limited key data provided. For further details on electrochemical methods reported for detection of mTBI biomarkers the readers could refer to the article from Pankratova *et al.* [60].

Abbreviations: 3-ICT: (3-Isocyanatopropyl)triethoxysilane; **4-ATP:** 4-aminothiophenol; **4-MBA:** 4-mercaptobenzoic acid; **Ab:** antibody; **AEDP:** monomer, 2-acrylamidoethyl dihydrogen phosphate; **AIBN:** 2,2'-azoisobutyronitrile; **ALP:** alkaline phosphatase; **ASV:** anodic stripping voltammetry; **Au:** gold electrode; **AuNCS:** gold nanoclasters; **CD:** carbon dots; **CD-GS:** β-cyclodextrin-graphene sheets; **CL:** chmiluminescence; **CLIA:** chemiluminescence immunoassay; **CM EU:** Carboxylate-modified polystyrene Eu(iii) chelate microparticles; **CS:** chitosan; **CSV:** cathodic stripping voltammetry; **DE:** disc electrode; **DMAA:** monomer, dimethylacrylamide; **DPASV:** differential pulse anodic stripping voltammetry; **DPM:** dipyrromethene; **DPTA:** thiol derivative of pentetic acid; **DTSP:** dithiobis (succinimidyl propionate); **DPV:** differential pulse voltammetry; **ECL:** electrochemiluminescence ; **EDC:** 1-ethyl-3-(3-dimethylaminopropyl) carbodiimide; **EDTA:** Ethylenediaminetetraacetic acid; **EGDMA:** ethylene glycol dimethylacryalte; **EIA:** enzyme immunoassay; **EIS:** electrochemical impedance spectroscopy; **ELISA:** enzyme linked immunosorbent assay ; **FED:** field-effect based detection (voltage controlled current amplification); **FEED:** field-effect enzymatic detection; **FRGG:** p-Nitrobenzene diazonium tetrafluoroborate (Fast Red GG salt); **FTO:** fluorine-doped tin oxide; **GA:** glutaraldehyde; **GCE:** glassy carbon electrode; **GNP:** gold nanoparticles; **GRONRs:** graphene oxide nanoribons; **HAuNPs:** hollow gold nanoparticles; **HRP:** horseradish peroxidase; **hSAM:** homogenous self-assembled monolayer; **IDE:** interdigitated electrode; **ITO:** indium tin oxide; **LFIA:** lateral flow immunoassay; **MB:** magnetic beads; **MBT:** 4-mercaptobutanol; **MCOH**: mercaptoethanol; **MDEA:** microdisc electrode array; **MECS:** macroelectrode with a comb structure; **MHDA:** mercaptohexadecanoic acid; **MIP:** molecularly imprinted polymer; **MOF:** metal-organic framework; **MPOH:** 3-mercaptopropanol; **mSAM:** mixed self-assembled monolayer; **MWCNTs:** multiwalled carbon nanotubes; **MUA:** 11-Mercaptoundecanoic acid; **NAC:** N-acetylcysteamine; N**HS:** N-hydroxysuccinimide; **NTCDI:** naphthalenetetracarboxylic diimide; **OFET:** organic field effect transistor; **OSWV:** Osteryoung square-wave voltammetry; **PAA:** polyacrylic acid; **PEC:** photo-electrochemical; **PEG:** polyethylene glycol; **PEI:** poly(ethyleneimine); **PICA:** poly (indole-5-carboxylic acid); **PMMA:** poly(methyl methacrylate); **PS**: polystyrene; **PS-MA:** polystyrene-co-methacrylic acid; **RAGE:** receptor domains for advanced glycation end products (three extracellular immunoglobulin domains: V, C1, C2); **rGRO:** reduced graphene oxide; **SAM(s):** self-assembled monolayer(s); **SDS:** sodium-dodecyl sulphate; **SERS:** surface enhanced Raman spectroscopy; **SFI:** single frequency impedance; **SPCE:** screen printed carbon electrode; **S-S:** disulfide bridge; **SWCNTs:** single-walled carbon nanotubes; **SWV:** square wave voltammetry; **T:** target; **TCEP:** tris (2-carboxyethyl) phosphine hydrochloride; **TCPP:** tetrakis (4-carboxyphenyl) porphyrin; **TFT:** thin film transistor; **TRF:** time-resolved fluorescence; **QCM:** quartz crystal microbalance; **QD:** quantum dot; **QLISA:** quantum dot-linked immunosorbent assays.

Figure S1: Four-parameter (4PL) dose-response nonlinear regression model for MSD calibration curves.

GFAP

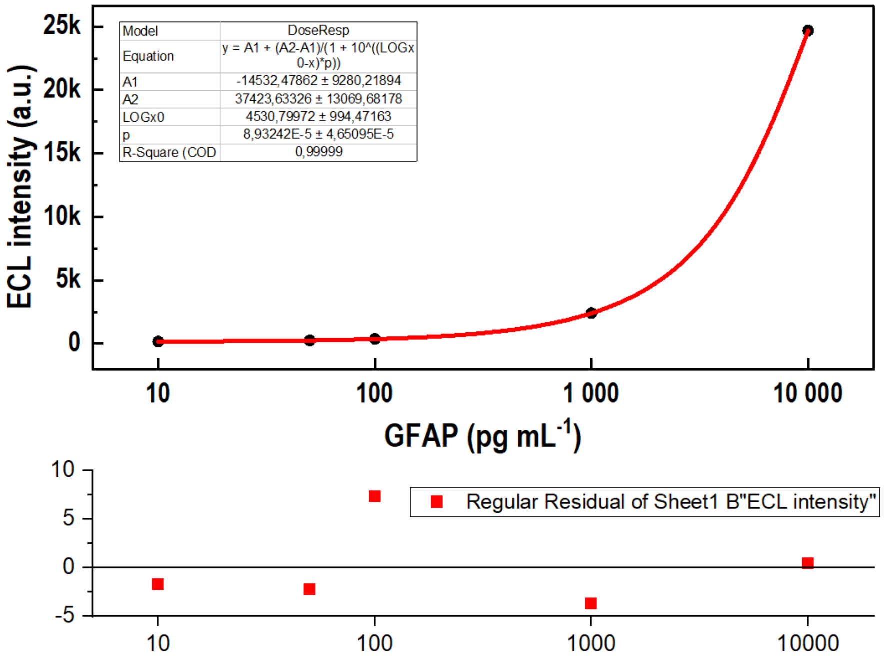

Parameters

		Value	Standard Error	t-Value	Prob>|t|	Dependency
ECL intensity	A1	-14532,47862	9280,21894	-1,56596	0,25785	1
	A2	37423,63326	13069,68178	2,86339	0,10339	1
	LOGx0	4530,79972	994,47163	4,55599	0,04495	1
	p	8,93242E-5	4,65095E-5	1,92056	0,19476	1
	span	51956,11188	22347,17582			
	EC20	0	0			
	EC50	--	--			
	EC80	--	--			

ANOVA

		DF	Sum of Squares	Mean Square	F Value	Prob>F
ECL intensity	Regression	4	28032,81068	7008,20267	74035,94387	1,35068E-5
	Residual	2	0,18932	0,09466		
	Uncorrected Total	6	28033			
	Corrected Total	5	26219,21729			

h-FABP

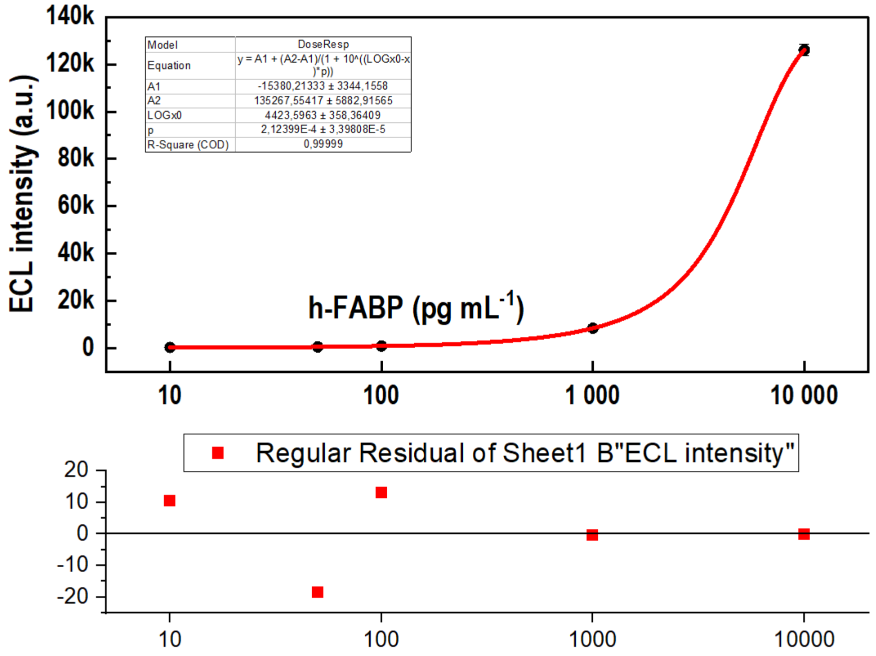

Parameters

		Value	Standard Error	t-Value	Prob>|t|	Dependency
ECL intensity	A1	-15380,21333	3344,1558	-4,59913	0,04417	0,99999
	A2	135267,55417	5882,91565	22,99328	0,00189	0,99987
	LOGx0	4423,5963	358,36409	12,34386	0,0065	0,99999
	p	2,12399E-4	3,39808E-5	6,25056	0,02465	1
	span	150647,76751	9217,74601			
	EC20	--	--			
	EC50	--	--			
	EC80	--	--			

ANOVA

		DF	Sum of Squares	Mean Square	F Value	Prob>F
ECL intensity	Regression	4	136120,8513	34030,21282	45923,536	2,1775E-5
	Residual	2	1,48204	0,74102		
	Uncorrected Total	6	136122,33333			
	Corrected Total	5	133666,65886			

S100β

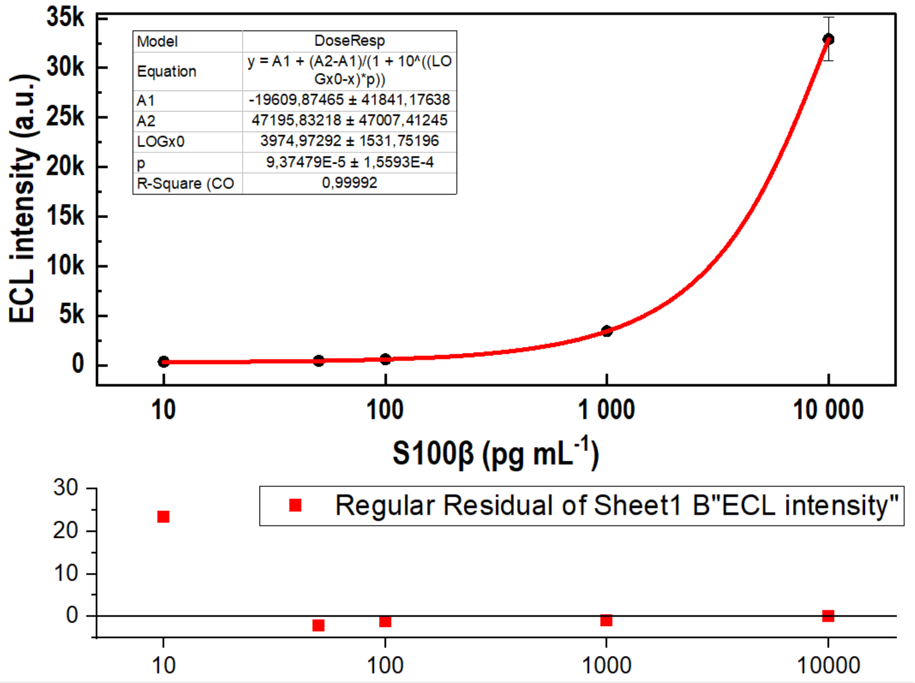

Parameters

		Value	Standard Error	t-Value	Prob>|t|	Dependency
ECL intensity	A1	-19609,87465	41841,17638	-0,46867	0,68542	1
	A2	47195,83218	47007,41245	1,00401	0,42111	1
	LOGx0	3974,97292	1531,75196	2,59505	0,12192	0,99999
	p	9,37479E-5	1,5593E-4	0,60122	0,60876	1
	span	66805,70684	88837,49438			
	EC20	0	0			
	EC50	--	--			
	EC80	--	--			

ANOVA

		DF	Sum of Squares	Mean Square	F Value	Prob>F
ECL intensity	Regression	4	37925,95577	9481,48894	6995,10675	1,42942E-4
	Residual	2	2,71089	1,35545		
	Uncorrected Total	6	37928,66667			
	Corrected Total	5	34692,76934			

Figure S1. Four-parameter (4PL) dose-response nonlinear regression model for MSD calibration curves.

Figure S2: Electrochemical characterization of commercially available Screen-Printed Electrodes (SPEs) for ECL applications.

(a)

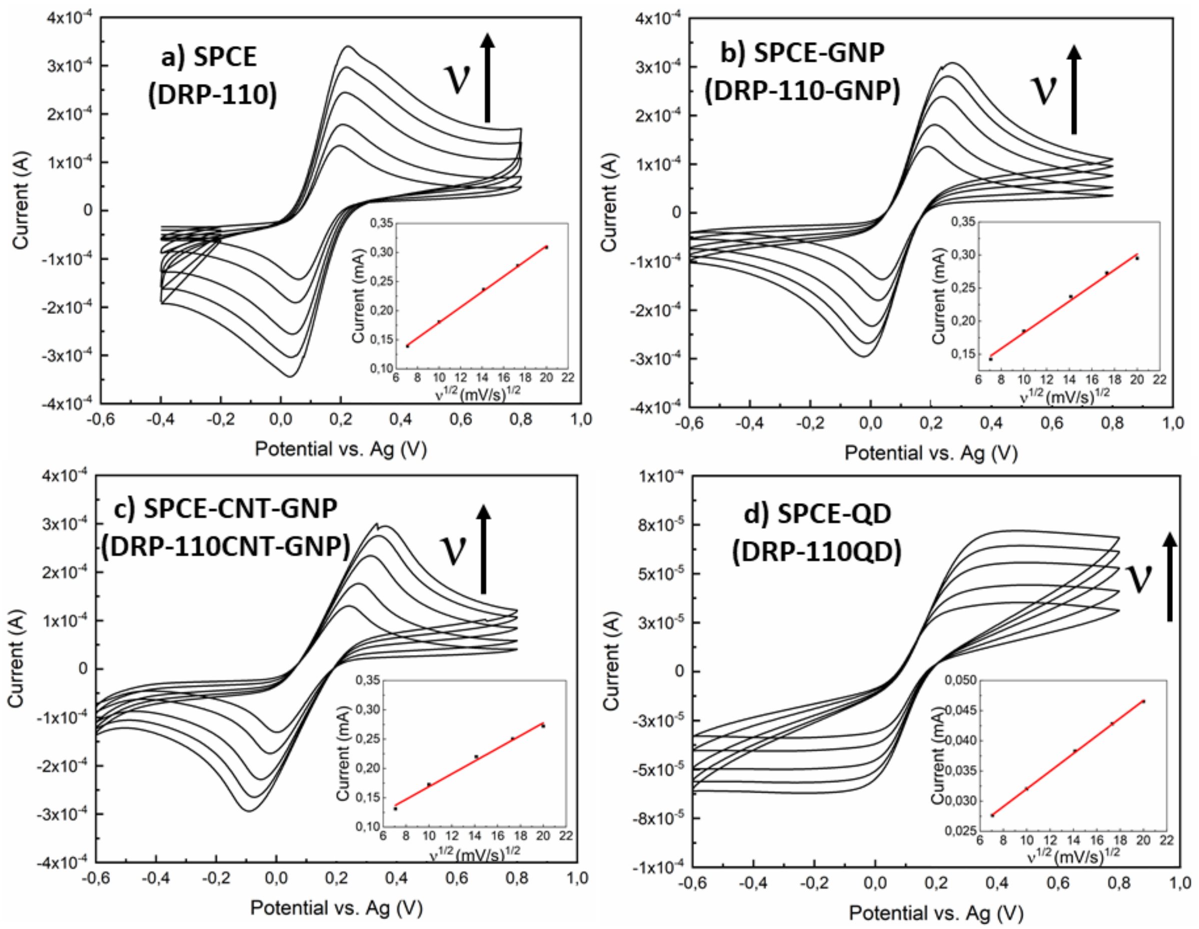

(b)

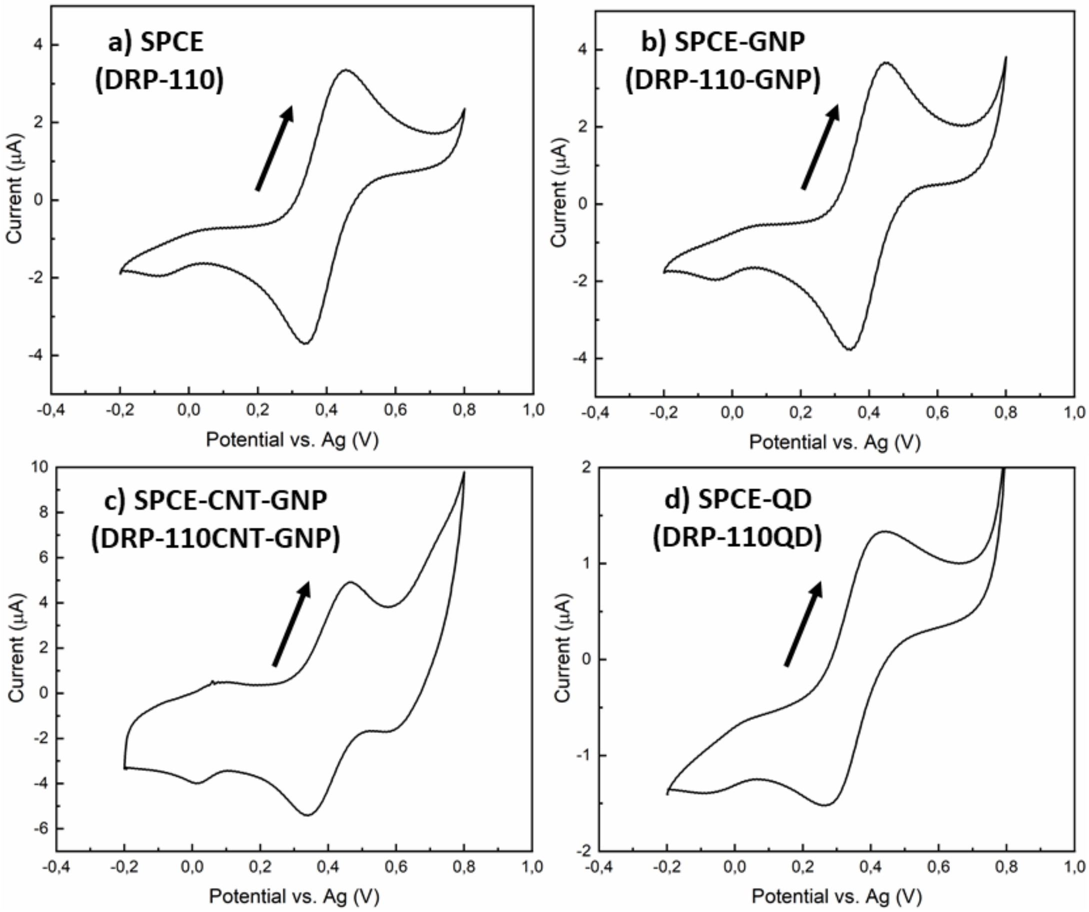

Figure S2. (a) Representative cyclic voltammograms of 5 mM $K_4[Fe(CN)_6]$/ $K_3[Fe(CN)_6]$ in PBS 1X (scan rates 50 mV/s, 100 mV/s, 200 mV/s, 300 mV/s and 400 mV/s) for: a) SPCE, b) SPCE-GNP, c) SPCE-CNT-GNP, d) SPCE-QD. Inset plot shows variation of peak current with scan rate, used to calculate the electroactive area of each electrode. **(b)** Representative cyclic voltammograms of 4 mM $[Ru(bpy)_3]^{2+}$ in PBS 1X (scan rate 0.1 V/s): a) SPCE (DRP-110), b) SPCE-GNP (DRP-110-GNP), c) SPCE-CNT-GNP (DRP-110CNT-GNP), d) SPCE-QD (DRP-110QD).

Figure S3: SEM images of commercially available Screen-Printed Carbon Electrodes (SPCE) for ECL applications.

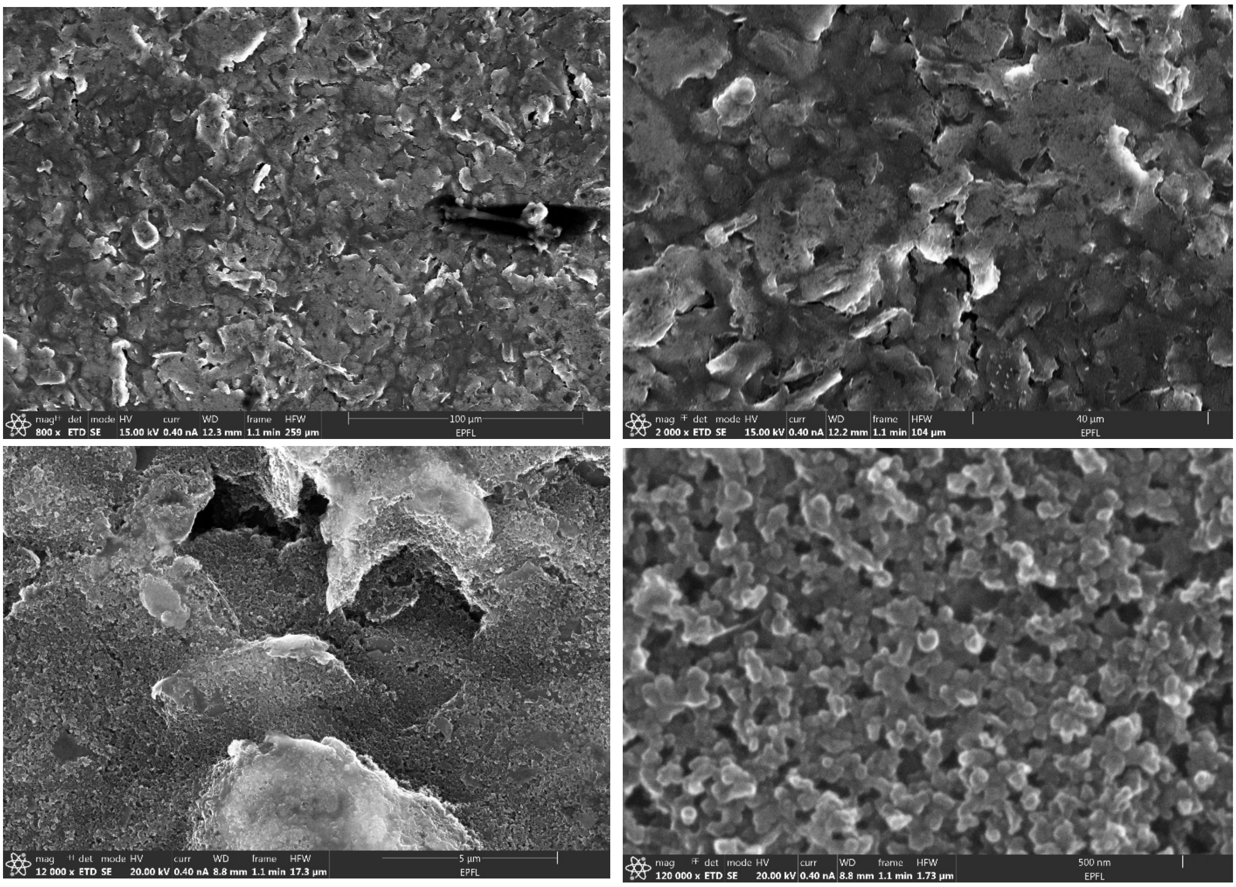

Figure S3. SEM images of SPCE (DRP-110) obtained with different magnifications (FEI Teneo scanning electron microscope (SEM) operated in high vacuum mode).

Figure S4: Linear regression model for SPCE µSTAT-ECL calibration curves.

GFAP

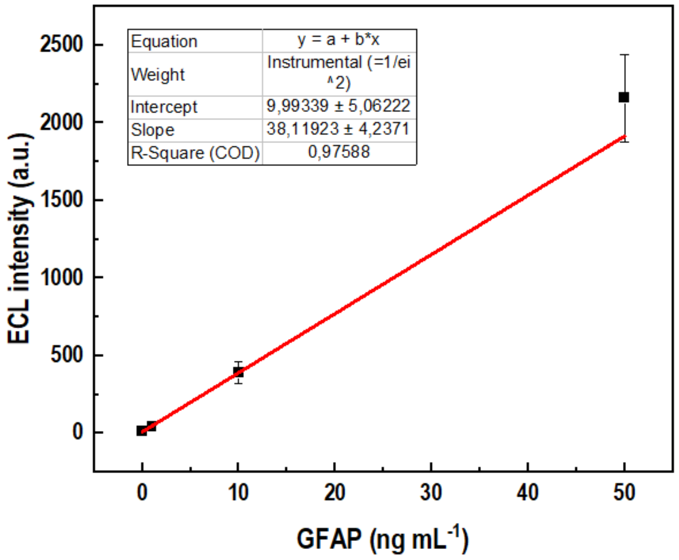

Parameters

		Value	Standard Error	t-Value	Prob>|t|
ECL intensity	Intercept	9,99339	5,06222	1,97411	0,18707
	Slope	38,11923	4,23716	8,99641	0,01213

Slope is significantly different from zero (See ANOVA Table).

Standard Error was scaled with square root of reduced Chi-Sqr.

Statistics

	ECL intensity
Number of Points	4
Degrees of Freedom	2
Residual Sum of Squares	2,2572
Pearson's r	0,98787
R Value	0,98787
R-Square (COD)	0,97588
Adj. R-Square	0,96383

Summary

	Intercept		Slope		Statistics	
	Value	Standard Error	Value	Standard Error	R-Square (COD)	Adj. R-Square
ECL intensity	9,99339	5,06222	38,11923	4,23716	0,97588	0,96383

ANOVA

		DF	Sum of Squares	Mean Square	F Value	Prob>F
ECL intensity	Model	1	91,34366	91,34366	80,93546	0,01213
	Error	2	2,2572	1,1286		
	Total	3	93,60086			

h-FABP

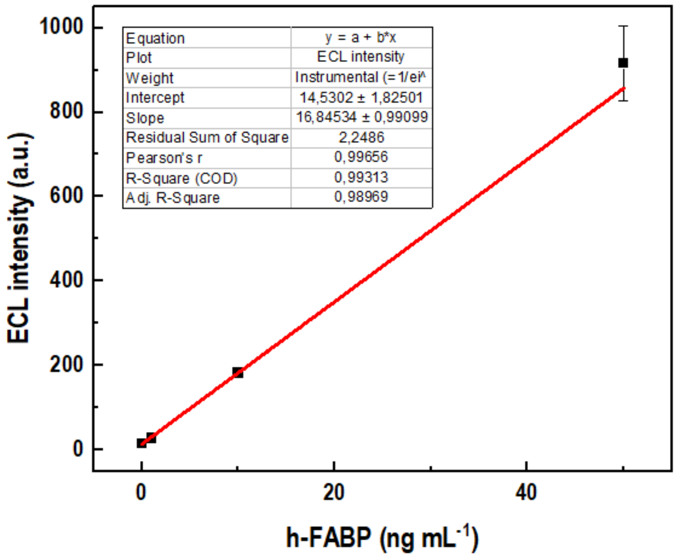

Parameters

		Value	Standard Error	t-Value	Prob>|t|
ECL intensity	Intercept	14,5302	1,82501	7,96171	0,01541
	Slope	16,84534	0,99099	16,99857	0,00344

Slope is significantly different from zero (See ANOVA Table).

Standard Error was scaled with square root of reduced Chi-Sqr.

Statistics

	ECL intensity
Number of Points	4
Degrees of Freedom	2
Residual Sum of Squares	2,2486
Pearson's r	0,99656
R-Square (COD)	0,99313
Adj. R-Square	0,98969

Summary

	Intercept		Slope		Statistics
	Value	Standard Error	Value	Standard Error	Adj. R-Square
ECL intensity	14,5302	1,82501	16,84534	0,99099	0,98969

ANOVA

		DF	Sum of Squares	Mean Square	F Value	Prob>F
ECL intensity	Model	1	324,86804	324,86804	288,95122	0,00344
	Error	2	2,2486	1,1243		
	Total	3	327,11664			

S100β

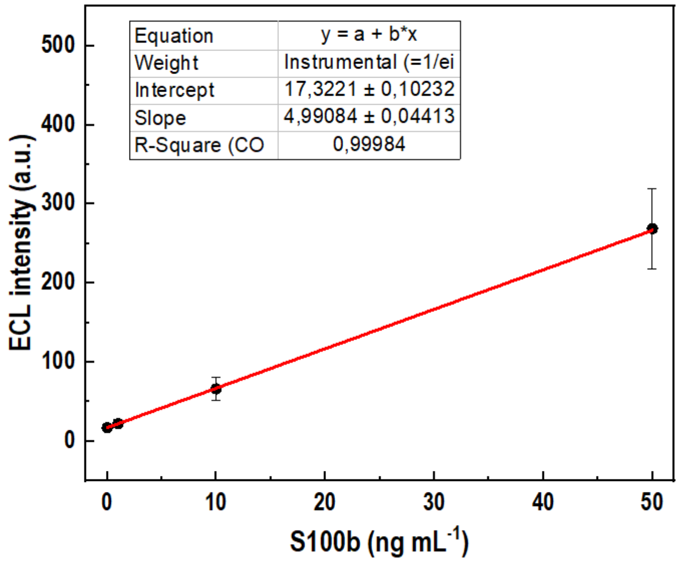

Parameters

		Value	Standard Error	t-Value	Prob>|t|
ECL intensity	Intercept	17,3221	0,10232	169,29125	3,48906E-5
	Slope	4,99084	0,04413	113,10552	7,81594E-5

Slope is significantly different from zero (See ANOVA Table).
Standard Error was scaled with square root of reduced Chi-Sqr.

Statistics

	ECL intensity
Number of Points	4
Degrees of Freedom	2
Residual Sum of Squares	0,00561
Pearson's r	0,99992
R Value	0,99992
R-Square (COD)	0,99984
Adj. R-Square	0,99977

Summary

	Intercept		Slope		Statistics	
	Value	Standard Error	Value	Standard Error	R-Square (COD)	Adj. R-Square
ECL intensity	17,3221	0,10232	4,99084	0,04413	0,99984	0,99977

ANOVA

		DF	Sum of Squares	Mean Square	F Value	Prob>F
ECL intensity	Model	1	35,86231	35,86231	12792,85835	7,81594E-5
	Error	2	0,00561	0,0028		
	Total	3	35,86792			

Figure S4. Linear regression model for SPCE µSTAT-ECL calibration curves.

Figure S5: Results of the recovery studies.

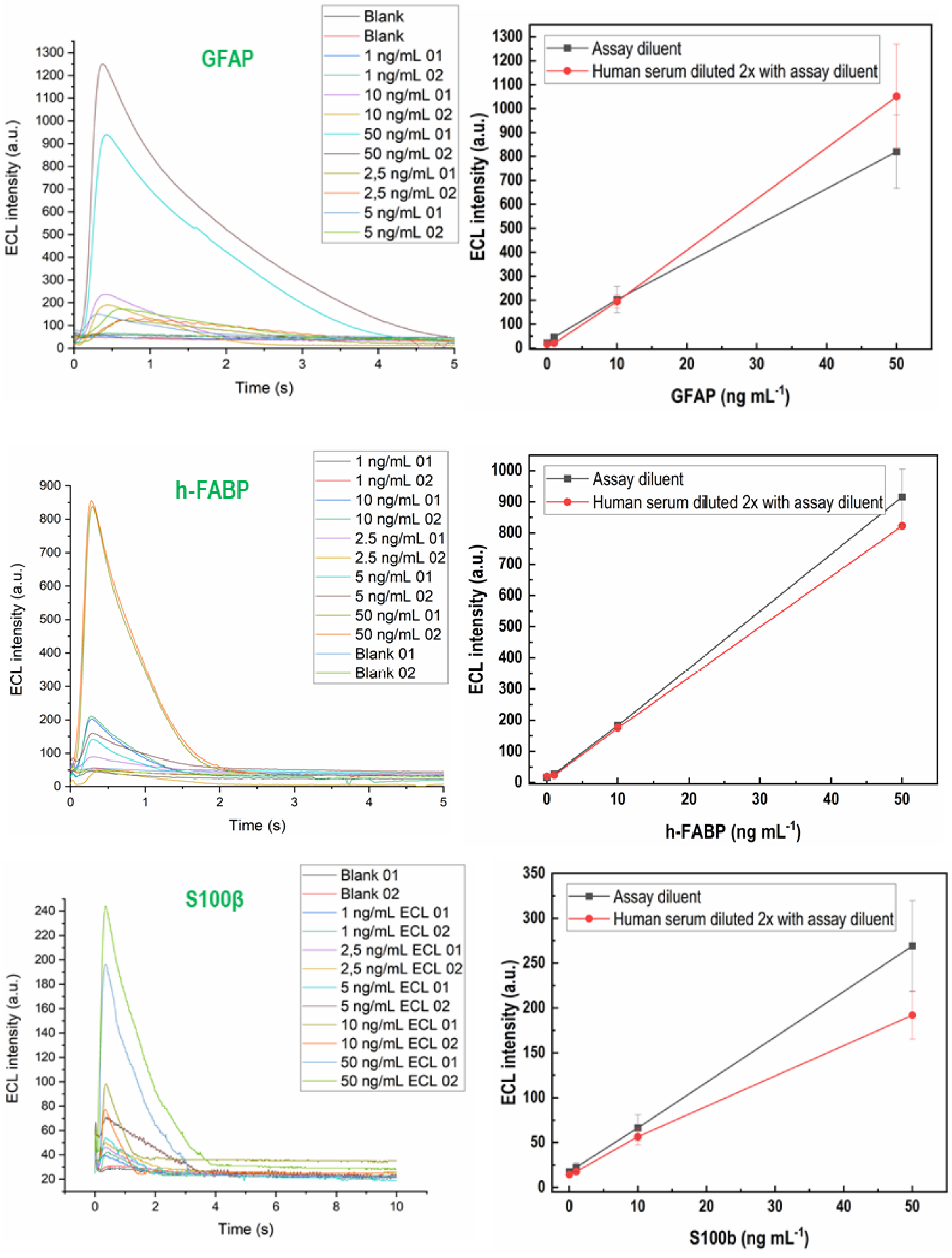

Figure S5. Left figures – chronoamperograms obtained for measurements of serum samples diluted 2x with the respective assay diluent and spiked with various concentrations of GFAP, h-FABP and S100β biomarkers (0 ng mL^{-1}, 1 ng mL^{-1}, 5 ng mL^{-1}, and 10 ng mL^{-1}) (n = 2, recoveries calculated for 2.5 ng mL^{-1} and 5 ng mL^{-1}, calculated recoveries in **Table 5**). **Right figures** – calibration curves for GFAP, h-FABP and S100β biomarkers obtained in each respective assay diluent (black line) and in serum samples diluted 2x with the respective assay diluent (red line).

Figure S6: CAD drawing of incubation cells used for SPEs.

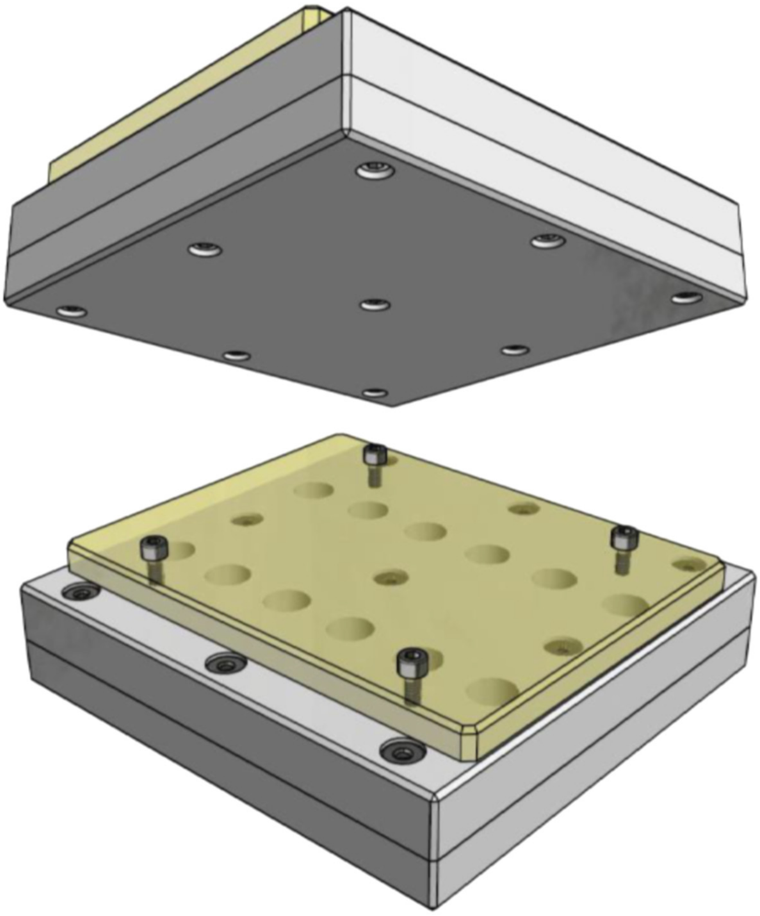

Figure S6. CAD drawing of customized incubation cell for SPEs fabricated at the HES-SO Valais-Wallis mechanical workshop for the Master Thesis project of Mr. Edis Saini at HES-SO entitled "*Conceptualization and development of a multiplex, high-sensitivity immunodiagnostic assay for neuropathologies*".

References

1. Arya, S.K.; Pui, T.S.; Wong, C.C.; Kumar, S.; Rahman, A.R.A. Effects of the Electrode Size and Modification Protocol on a Label-Free Electrochemical Biosensor. *Langmuir* **2013**, *29*, 6770–6777.
2. Huang, W.; Besar, K.; LeCover, R.; Dulloor, P.; Sinha, J.; Martínez Hardigree, J.F.; Pick, C.; Swavola, J.; Everett, A.D.; Frechette, J.; et al. Label-Free Brain Injury Biomarker Detection Based on Highly Sensitive Large Area Organic Thin Film Transistor with Hybrid Coupling Layer. *Chem. Sci.* **2014**, *5*, 416–426.
3. Wang, T.; Fang, Y.; He, Z. Electrochemical Quantitative Detection of Glial Fibrillary Acidic Protein Based on Molecularly Imprinted Polymer Sensor. *INTERNATIONAL JOURNAL OF ELECTROCHEMICAL SCIENCE* **2017**, *12*, 7341–7350.
4. Song, J.; Dailey, J.; Li, H.; Jang, H.-J.; Zhang, P.; Wang, J.T.-H.; Everett, A.D.; Katz, H.E. Extended Solution Gate OFET-Based Biosensor for Label-Free Glial Fibrillary Acidic Protein Detection with Polyethylene Glycol-Containing Bioreceptor Layer. *Advanced Functional Materials* **2017**, *27*, 1606506 1-7.
5. Khetani, S.; Ozhukil Kollath, V.; Kundra, V.; Nguyen, M.D.; Debert, C.; Sen, A.; Karan, K.; Sanati-Nezhad, A. Polyethylenimine Modified Graphene-Oxide Electrochemical Immunosensor for the Detection of Glial Fibrillary Acidic Protein in Central Nervous System Injury. *ACS sensors* **2018**, *3*, 844–851.
6. Ma, Y.; Xu, G.; Wei, F.; Cen, Y.; Song, Y.; Ma, Y.; Xu, X.; Shi, M.; Sohail, M.; Hu, Q. Carbon Dots Based Immunosorbent Assay for the Determination of GFAP in Human Serum. *Nanotechnology* **2018**, *29*, 145501, doi:10.1088/1361-6528/aaabea.
7. Cardinell, B.A.; Addington, C.P.; Stabenfeldt, S.E.; La Belle, J.T. Multi-Biomarker Detection Following Traumatic Brain Injury. *Critical Reviews™ in Biomedical Engineering* **2019**, *47*, 193–206.
8. Agostini, M.; Amato, F.; Vieri, M.L.; Greco, G.; Tonazzini, I.; Baroncelli, L.; Caleo, M.; Vannini, E.; Santi, M.; Signore, G.; et al. Glial-Fibrillary-Acidic-Protein (GFAP) Biomarker Detection in Serum-Matrix: Functionalization Strategies and Detection by an Ultra-High-Frequency Surface-Acoustic-Wave (UHF-SAW) Lab-on-Chip. *Biosensors and Bioelectronics* **2021**, *172*, 112774, doi:10.1016/j.bios.2020.112774.
9. Button, E.B.; Cheng, W.H.; Barron, C.; Cheung, H.; Bashir, A.; Cooper, J.; Gill, J.; Stukas, S.; Baron, D.C.; Robert, J.; et al. Development of a Novel, Sensitive Translational Immunoassay to Detect Plasma Glial Fibrillary Acidic Protein (GFAP) after Murine Traumatic Brain Injury. *Alz Res Therapy* **2021**, *13*, 58, doi:10.1186/s13195-021-00793-9.
10. Krausz, A.D.; Korley, F.K.; Burns, M.A. A Variable Height Microfluidic Device for Multiplexed Immunoassay Analysis of Traumatic Brain Injury Biomarkers. *Biosensors* **2021**, *11*, 320, doi:10.3390/bios11090320.
11. Natarajan, S.; Joseph, J. A Novel Time-Resolved Fluorescent Lateral Flow Immunoassay for Quantitative Detection of the Trauma Brain Injury Biomarker-Glial Fibrillary Acidic Protein. *Sens. Diagn.* **2022**, *1*, 193–197, doi:10.1039/D1SD00021G.
12. GFAP ELISA Kit | Sigma-Aldrich Available online: http://www.sigmaaldrich.com/ (accessed on 30 August 2021).
13. R-PLEX Human GFAP Antibody Set | Meso Scale Discovery Available online: https://www.mesoscale.com/en/products/r-plex-human-gfap-antibody-set-f211m/ (accessed on 31 August 2021).
14. Https://Www.Banyanbio.Com/Assets/Files/000175_vA-IFU-Banyan-BTI.Pdf.
15. Bazarian, J.J.; Biberthaler, P.; Welch, R.D.; Lewis, L.M.; Barzo, P.; Bogner-Flatz, V.; Brolinson, P.G.; Büki, A.; Chen, J.Y.; Christenson, R.H.; et al. Serum GFAP and UCH-L1 for Prediction of Absence of Intracranial Injuries on Head CT (ALERT-TBI): A Multicentre Observational Study. *The Lancet Neurology* **2018**, *17*, 782–789.
16. Simoa Technology, Https://Www.Quanterix.Com/Technology, Last Accessed 27.04.2020. *Quanterix* 2019.
17. TBI Plasma Cartridge | Abbott Point of Care Available online: https://www.pointofcare.abbott/us/en/offerings/istat/istat-test-cartridges/TBI-Plasma (accessed on 30 August 2021).
18. Okonkwo, D.O.; Puffer, R.C.; Puccio, A.M.; Yuh, E.L.; Yue, J.K.; Diaz-Arrastia, R.; Korley, F.K.; Wang, K.K.W.; Sun, X.; Taylor, S.R.; et al. Point-of-Care Platform Blood Biomarker Testing of Glial Fibrillary Acidic Protein versus S100 Calcium-Binding Protein B for Prediction of Traumatic Brain Injuries: A Transforming Research and Clinical Knowledge in Traumatic Brain Injury Study. *J Neurotrauma* **2020**, *37*, 2460–2467, doi:10.1089/neu.2020.7140.
19. Korley, F.K.; Datwyler, S.A.; Jain, S.; Sun, X.; Beligere, G.; Chandran, R.; Marino, J.A.; McQuiston, B.; Zhang, H.; Caudle, K.L.; et al. Comparison of GFAP and UCH-L1 Measurements from Two Prototype Assays: The Abbott i-STAT and ARCHITECT Assays. *Neurotrauma Rep* **2021**, *2*, 193–199, doi:10.1089/neur.2020.0037.
20. Miller, C.J.; Hu, J. Magnetic Immunosensor and Method of Use 2018.

21. biodirection_zmuop8 The NanoDx^{TM} System for TBI | Our Solution Available online: https://nanodiagnostics.com/our-solution/the-nanodx-system-for-tbi/ (accessed on 30 August 2021).
22. Feng, L.-N.; Bian, Z.-P.; Peng, J.; Jiang, F.; Yang, G.-H.; Zhu, Y.-D.; Yang, D.; Jiang, L.-P.; Zhu, J.-J. Ultrasensitive Multianalyte Electrochemical Immunoassay Based on Metal Ion Functionalized Titanium Phosphate Nanospheres. *Anal. Chem.* **2012**, *84*, 7810–7815.
23. Stan, D.; Mihailescu, C.-M.; Iosub, R.; Moldovan, C.; Savin, M.; Baciu, I. Electrochemical Studies of Homogeneous Self-Assembled Monolayers versus Mixed Self-Assembled Monolayers on Gold Electrode for “Label Free” Detection of Heart Fatty Acid Binding Protein. *Thin Solid Films* **2012**, *526*, 143–149.
24. Carless, D.R.; Wnęk, M.; Knox, C.; Harrison, K.R.; Calder, N.; Hall, A.S.; Barth, J.H. Clinical and Analytical Evaluation of an Immunoturbidimetric Heart-Type Fatty Acid-Binding Protein Assay. *Scandinavian Journal of Clinical and Laboratory Investigation* **2013**, *73*, 48–53, doi:10.3109/00365513.2012.734396.
25. Mihailescu, C.-M.; Stan, D.; Iosub, R.; Moldovan, C.; Savin, M. A Sensitive Capacitive Immunosensor for Direct Detection of Human Heart Fatty Acid-Binding Protein (h-FABP). *Talanta* **2015**, *132*, 37–43.
26. Qin, X.; Xu, A.; Liu, L.; Sui, Y.; Li, Y.; Tan, Y.; Chen, C.; Xie, Q. Selective Staining of CdS on ZnO Biolabel for Ultrasensitive Sandwich-Type Amperometric Immunoassay of Human Heart-Type Fatty-Acid-Binding Protein and Immunoglobulin G. *Biosensors and Bioelectronics* **2017**, *91*, 321–327.
27. Qin, X.; Zhang, X.; Wang, M.; Dong, Y.; Liu, J.; Zhu, Z.; Li, M.; Yang, D.; Shao, Y. Fabrication of Tris(Bipyridine)Ruthenium(II)-Functionalized Metal–Organic Framework Thin Films by Electrochemically Assisted Self-Assembly Technique for Electrochemiluminescent Immunoassay. *Anal. Chem.* **2018**, *90*, 11622–11628.
28. Savin, M.; Mihailescu, C.-M.; Matei, I.; Stan, D.; Moldovan, C.A.; Ion, M.; Baciu, I. A Quantum Dot-Based Lateral Flow Immunoassay for the Sensitive Detection of Human Heart Fatty Acid Binding Protein (HFABP) in Human Serum. *Talanta* **2018**, *178*, 910–915, doi:10.1016/j.talanta.2017.10.045.
29. Crapnell, R.D.; Canfarotta, F.; Czulak, J.; Johnson, R.; Betlem, K.; Mecozzi, F.; Down, M.P.; Eersels, K.; van Grinsven, B.; Cleij, T.J.; et al. Thermal Detection of Cardiac Biomarkers Heart-Fatty Acid Binding Protein and ST2 Using a Molecularly Imprinted Nanoparticle-Based Multiplex Sensor Platform. *ACS Sens.* **2019**, *4*, 2838–2845, doi:10.1021/acssensors.9b01666.
30. Li, F.; Guo, L.; Hu, Y.; Li, Z.; Liu, J.; He, J.; Cui, H. Multiplexed Chemiluminescence Determination of Three Acute Myocardial Infarction Biomarkers Based on Microfluidic Paper-Based Immunodevice Dual Amplified by Multifunctionalized Gold Nanoparticles. *Talanta* **2020**, *207*, 120346, doi:10.1016/j.talanta.2019.120346.
31. Gan, X.; Han, D.; Wang, J.; Liu, P.; Li, X.; Zheng, Q.; Yan, Y. A Highly Sensitive Electrochemiluminescence Immunosensor for H-FABP Determination Based on Self-Enhanced Luminophore Coupled with Ultrathin 2D Nickel Metal-Organic Framework Nanosheets. *Biosens Bioelectron* **2021**, *171*, 112735, doi:10.1016/j.bios.2020.112735.
32. TBICheck - The Fastest Diagnostic Test for Traumatic Brain Injury Available online: https://tbicheck.com/home (accessed on 30 August 2021).
33. Human H-FABP ELISA Kit (Ab243682) | Abcam Available online: https://www.abcam.com/Human-H-FABP-ELISA-Kit-ab243682.html?gclsrc=aw.ds|aw.ds&gclid=Cj0KCQjwg7KJBhDyARIsAHrAXaH7SK6R4I_1DbOvLN1pxlnqW42hdrT0Nk4Yjew7t-ynxwT-aoeUn4EaAuTJEALw_wcB (accessed on 30 August 2021).
34. R-PLEX Human FABP3/H-FABP Antibody Set | Meso Scale Discovery Available online: https://www.mesoscale.com/en/products/r-plex-human-fabp3-h-fabp-antibody-set-f214t/ (accessed on 31 August 2021).
35. Missler, U.; Wiesmann, M.; Friedrich, C.; Kaps, M. S-100 Protein and Neuron-Specific Enolase Concentrations in Blood as Indicators of Infarction Volume and Prognosis in Acute Ischemic Stroke. *Stroke* **1997**, *28*, 1956–1960, doi:10.1161/01.STR.28.10.1956.
36. PII: S0022-1759(97)00050-1 | Elsevier Enhanced Reader Available online: https://reader.elsevier.com/reader/sd/pii/S0022175997000501?token=A464853B1ED8E1A9E89C9B8F041BB4E0DF5188385648EF4C35723E513BFC3977CFC76D86BA77D9A85AFD7B71FF48711E&originRegion=eu-west-1&originCreation=20210827152016 (accessed on 27 August 2021).
37. Leite, M.C.; Galland, F.; Brolese, G.; Guerra, M.C.; Bortolotto, J.W.; Freitas, R.; Almeida, L.M.V. de; Gottfried, C.; Gonçalves, C.-A. A Simple, Sensitive and Widely Applicable ELISA for S100B: Methodological Features of the Measurement of This Glial Protein. *Journal of Neuroscience Methods* **2008**, *169*, 93–99, doi:10.1016/j.jneumeth.2007.11.021.

38. Liu, Y.; Wang, H.; Chen, J.; Liu, C.; Li, W.; Kong, J.; Yang, P.; Liu, B. A Sensitive Microchip-Based Immunosensor for Electrochemical Detection of Low-Level Biomarker S100B. *Electroanalysis* **2013**, *25*, 1050–1055.
39. Wang, X.; Li, H.; Li, X.; Chen, Y.; Yin, Y.; Li, G. Electrochemical Assay of Melanoma Biomarker in Human Blood. *Electrochemistry Communications* **2014**, *39*, 12–14.
40. Mikuła, E.; Wysłouch-Cieszyńska, A.; Zhukova, L.; Puchalska, M.; Verwilst, P.; Dehaen, W.; Radecki, J.; Radecka, H. Voltammetric Detection of S100B Protein Using His-Tagged Receptor Domains for Advanced Glycation End Products (RAGE) Immobilized onto a Gold Electrode Surface. *Sensors* **2014**, *14*, 10650–10663.
41. Kim, C.; Searson, P.C. Magnetic Bead-Quantum Dot Assay for Detection of a Biomarker for Traumatic Brain Injury. *Nanoscale* **2015**, *7*, 17820–17826, doi:10.1039/C5NR05608J.
42. Kurzątkowska, K.; Jankowska, A.; Wysłouch-Cieszyńska, A.; Zhukova, L.; Puchalska, M.; Dehaen, W.; Radecka, H.; Radecki, J. Voltammetric Detection of the S100B Protein Using His-Tagged RAGE Domain Immobilized onto a Gold Electrode Modified with a Dipyrromethene–Cu(II) Complex and Different Diluents. *Journal of Electroanalytical Chemistry* **2016**, *767*, 76–83.
43. Khetani, S.; Aburashed, R.; Singh, A.; Sen, A.; Sanati-Nezhad, A. Immunosensing of S100β Biomarker for Diagnosis of Spinal Cord Injuries (SCI). *Sensors and Actuators B: Chemical* **2017**, *247*, 163–169.
44. Kuo, Y.-C.; Lee, C.-K.; Lin, C.-T. Improving Sensitivity of a Miniaturized Label-Free Electrochemical Biosensor Using Zigzag Electrodes. *Biosensors and Bioelectronics* **2018**, *103*, 130–137.
45. Wang, Y.; Zhao, P.; Mao, L.; Hou, Y.; Li, D. Determination of Brain Injury Biomarkers by Surface-Enhanced Raman Scattering Using Hollow Gold Nanospheres. *RSC Adv.* **2018**, *8*, 3143–3150, doi:10.1039/C7RA12410D.
46. Mathew, A.S.; Shi, X.; Yau, S.-T. Detection of a Traumatic Brain Injury Biomarker at the 10 Fg/ML Level. *Molecular Diagnosis & Therapy* **2018**, *22*, 729–735.
47. Sun, J.; Zhao, Y.; Hou, Y.; Li, H.; Yang, M.; Wang, Y.; Sun, B. Multiplexed Electrochemical and SERS Dual-Mode Detection of Stroke Biomarkers: Rapid Screening with High Sensitivity. *New Journal of Chemistry* **2019**, *43*, 13381–13387.
48. Tabrizi, M.A.; Ferré-Borrull, J.; Kapruwan, P.; Marsal, L.F. A Photoelectrochemical Sandwich Immunoassay for Protein S100β, a Biomarker for Alzheimer's Disease, Using an ITO Electrode Modified with a Reduced Graphene Oxide-Gold Conjugate and CdS-Labeled Secondary Antibody. *Microchimica Acta* **2019**, *186*, 117 1-9.
49. Harpaz, D.; Koh, B.; Marks, R.S.; Seet, R.C.S.; Abdulhalim, I.; Tok, A.I.Y. Point-of-Care Surface Plasmon Resonance Biosensor for Stroke Biomarkers NT-ProBNP and S100β Using a Functionalized Gold Chip with Specific Antibody. *Sensors* **2019**, *19*, 2533, doi:10.3390/s19112533.
50. Han, L.; Ding, C.; Guo, Y.; Wang, Y.; Ding, Y. Sensitively Detecting MTBI Biomarker S100B by Using Peptide-Modified Ratiometric Fluorescent C/AuNCs Nanoprobe. *Anal Bioanal Chem* **2020**, *412*, 3695–3702, doi:10.1007/s00216-020-02613-9.
51. Hassanain, W.A.; Sivanesan, A.; Ayoko, G.A.; Izake, E.L. Rapid Electrochemical Nanosensing of S100ß in Blood. *J. Electrochem. Soc.* **2020**, *167*, 067518, doi:10.1149/1945-7111/ab82fb.
52. Gao, X.; Boryczka, J.; Zheng, P.; Kasani, S.; Yang, F.; Engler-Chiurazzi, E.B.; Simpkins, J.W.; Wigginton, J.G.; Wu, N. A "Hot Spot"-Enhanced Paper Lateral Flow Assay for Ultrasensitive Detection of Traumatic Brain Injury Biomarker S-100β in Blood Plasma. *Biosensors and Bioelectronics* **2021**, *177*, 112967, doi:10.1016/j.bios.2021.112967.
53. Elecsys® S100 Available online: https://diagnostics.roche.com/global/en/products/params/elecsys-s100.html (accessed on 27 August 2021).
54. Delefortrie, Q.; Lejeune, F.; Kerzmann, B.; Levy, R.; Adam, J.-F.; Sottiaux, T.; Grimmelprez, A.; Vankerkhoven, P.; Hachimi-Idrissi, S. Evaluation of the Roche® Elecsys and the Diasorin® Liaison S100 Kits in the Management of Mild Head Injury in the Emergency Room. *Clinical Biochemistry* **2018**, *52*, 123–130, doi:10.1016/j.clinbiochem.2017.11.004.
55. CanAg® S100 EIA | Fujirebio Available online: https://www.fujirebio.com/en/products-solutions/canag-s100-eia (accessed on 27 August 2021).
56. Human S100B ELISA, EZHS100B-33K This Human S100B ELISA Kit Is to Be Used for the Quantification of Human S100B in Cerebrospinal Fluid, Serum & Plasma. | Sigma-Aldrich Available online: http://www.sigmaaldrich.com/ (accessed on 30 August 2021).
57. S100B ELISA Kit (Ab234573) | Abcam Available online: https://www.abcam.com/s100b-elisa-kit-ab234573.html (accessed on 30 August 2021).

58. Human S100B ELISA Kit by Bioss, Cat. No. BSKH1066 | Lucerna-Chem AG Available online: https://lucerna-chem.ch/shop/3438089/human-s100b-elisa-kit (accessed on 30 August 2021).
59. S100b ELISA Kit (Rabbit) (GWB-KBBVZ1) | Quantitative Sandwich ELISA Available online: https://www.genwaybio.com/s100b-elisa-kit-rabbit-gwb-kbbvz1 (accessed on 30 August 2021).
60. Pankratova, N.; Jović, M.; Pfeifer, M.E. Electrochemical Sensing of Blood Proteins for Mild Traumatic Brain Injury (MTBI) Diagnostics and Prognostics: Towards a Point-of-Care Application. *RSC Adv.* **2021**, *11*, 17301–17319, doi:10.1039/D1RA00589H.
